# Supplementary figures and images for: Optimizing canine T cell activation, expansion, and transduction
Source: PLoS One. 2025 Sep 11;20(9):e0324403. doi: 10.1371/journal.pone.0324403 (PMC12425254; doi:10.1371/journal.pone.0324403)

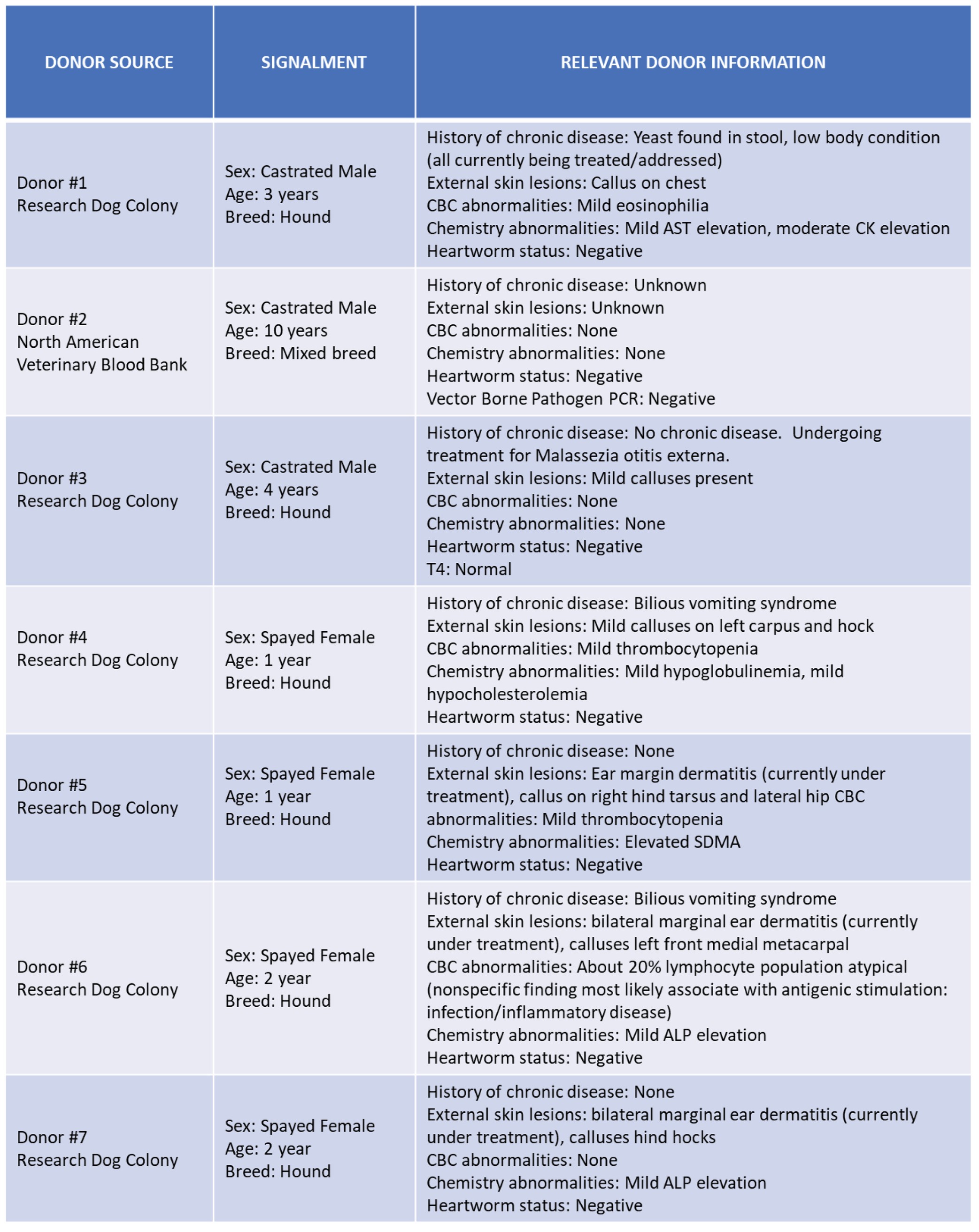

Supplement: S1 Table — A summary of pertinent medical information for the seven canine blood donors used in this study. (TIF) [file pone.0324403.s001.tif]

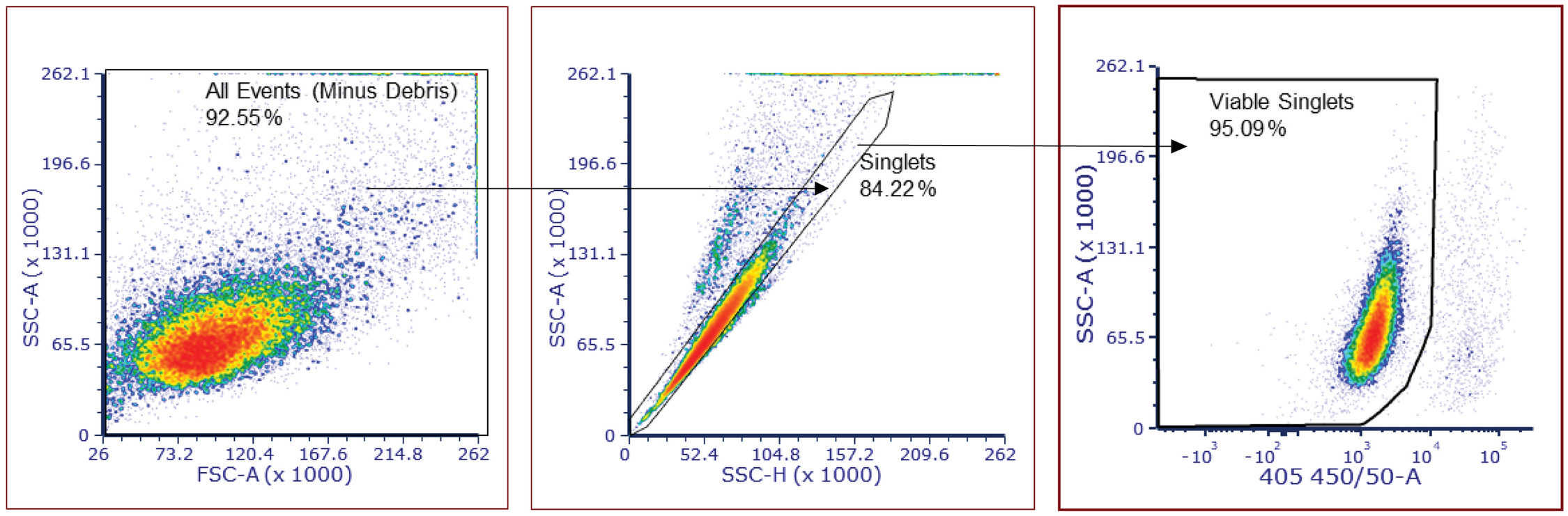

Supplement: S1 Fig — Representative flow cytometry dot plot highlighting gating strategy used to determine cell viability. The parent gate includes all events with the exclusion of debris, followed by gating on singlets, followed by gating on viable cells as indicated by the exclusion of viability dye. (TIF) [file pone.0324403.s002.tif]

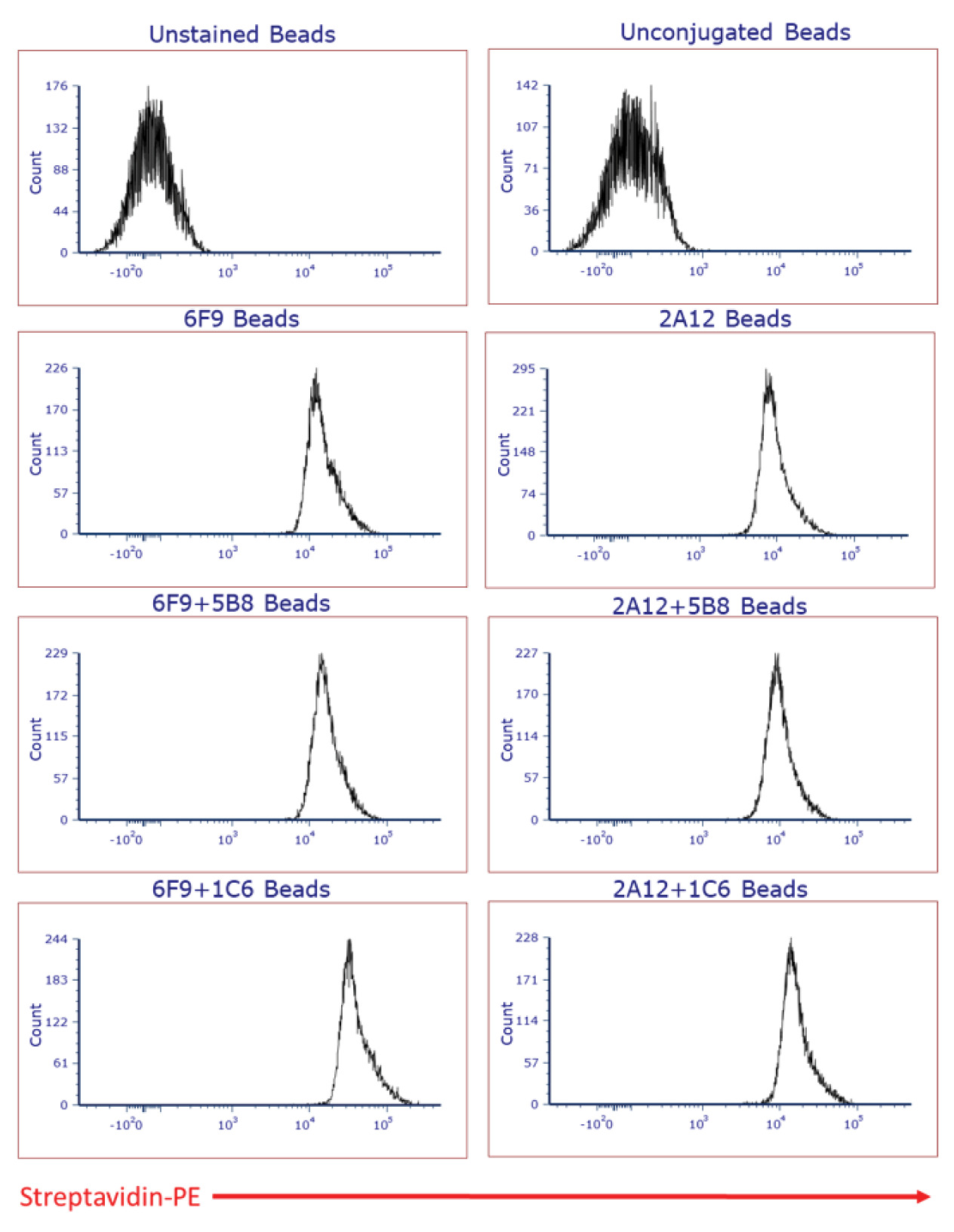

Supplement: S2 Fig — MACSiBeads contain anti-biotin antibody that will bind to biotinylated stimulatory antibody during the conjugation process. Beads were stained using streptavidin-PE to label biotinylated antibody on the surface of successfully conjugated beads. The unstained sample contains no streptavidin and was used as a negative control. Unconjugated beads contain no stimulatory antibody and thus show no PE signaling. All other beads contain stimulatory antibody and show PE signaling demonstrating successful conjugation. (TIF) [file pone.0324403.s003.tif]

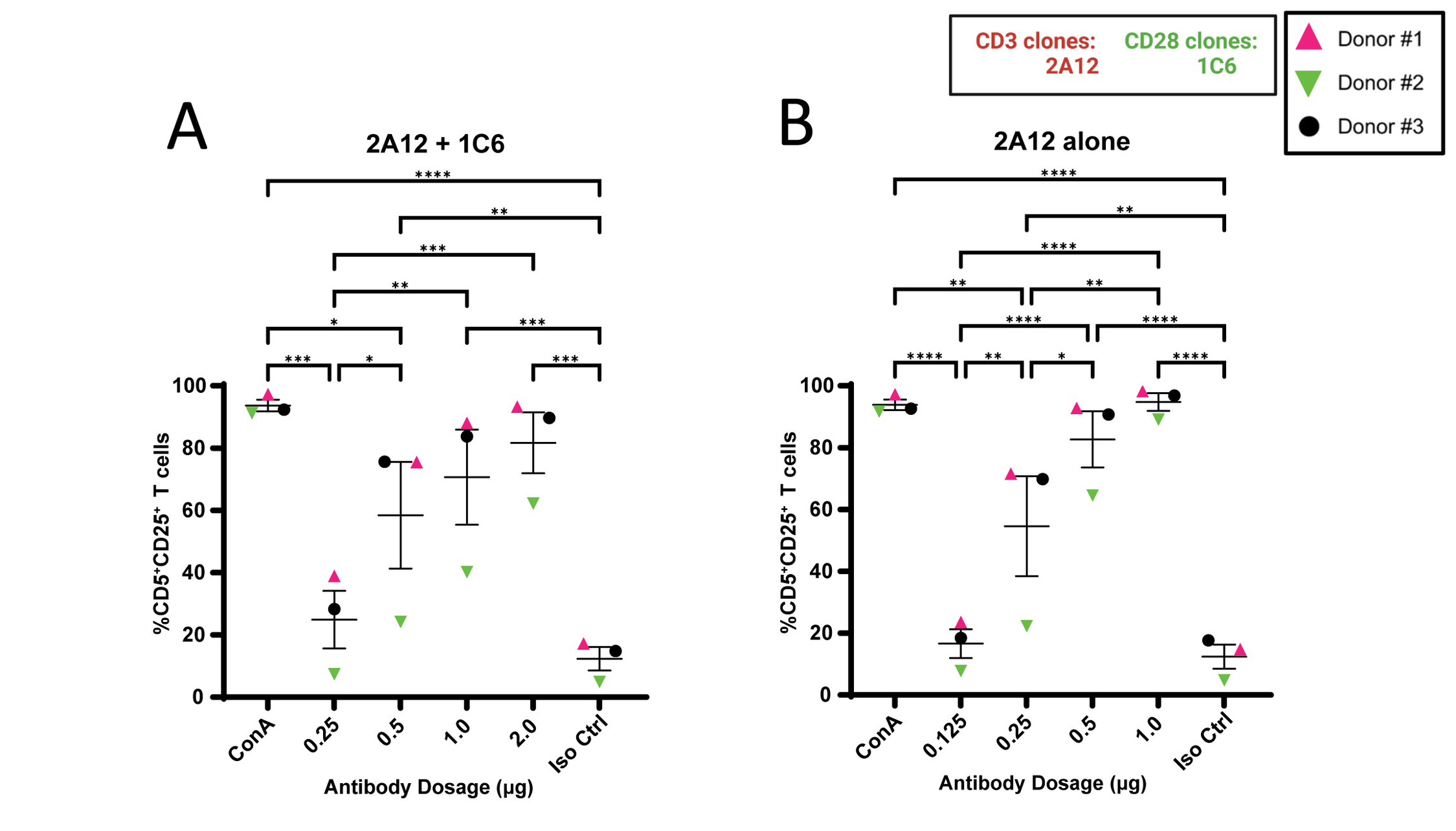

Supplement: S3 Fig — (A-B) This experiment was performed to confirm the findings in Fig 3 that demonstrated that plate-bound αCD28 clone 1C6 reduced activation efficiency in T cells when used (A) in combination with αCD3 clone 2A12 compared to stimulation with (B) 2A12 alone (2A12=CA17.2A12). Plate-bound stimulatory antibodies were used to activate PBMCs (n = 3) and frequency of CD5+ T cell activation was evaluated by cell surface expression of CD25. Horizontal lines indicate mean values, and error bars represent standard error of the mean. Pairwise statistical analysis was performed by One-way ANOVA with Tukey’s multiple means comparison; * p < 0.05, ** p < 0.01, *** p < 0.001, **** p < 0.0001. (TIF) [file pone.0324403.s004.tif]

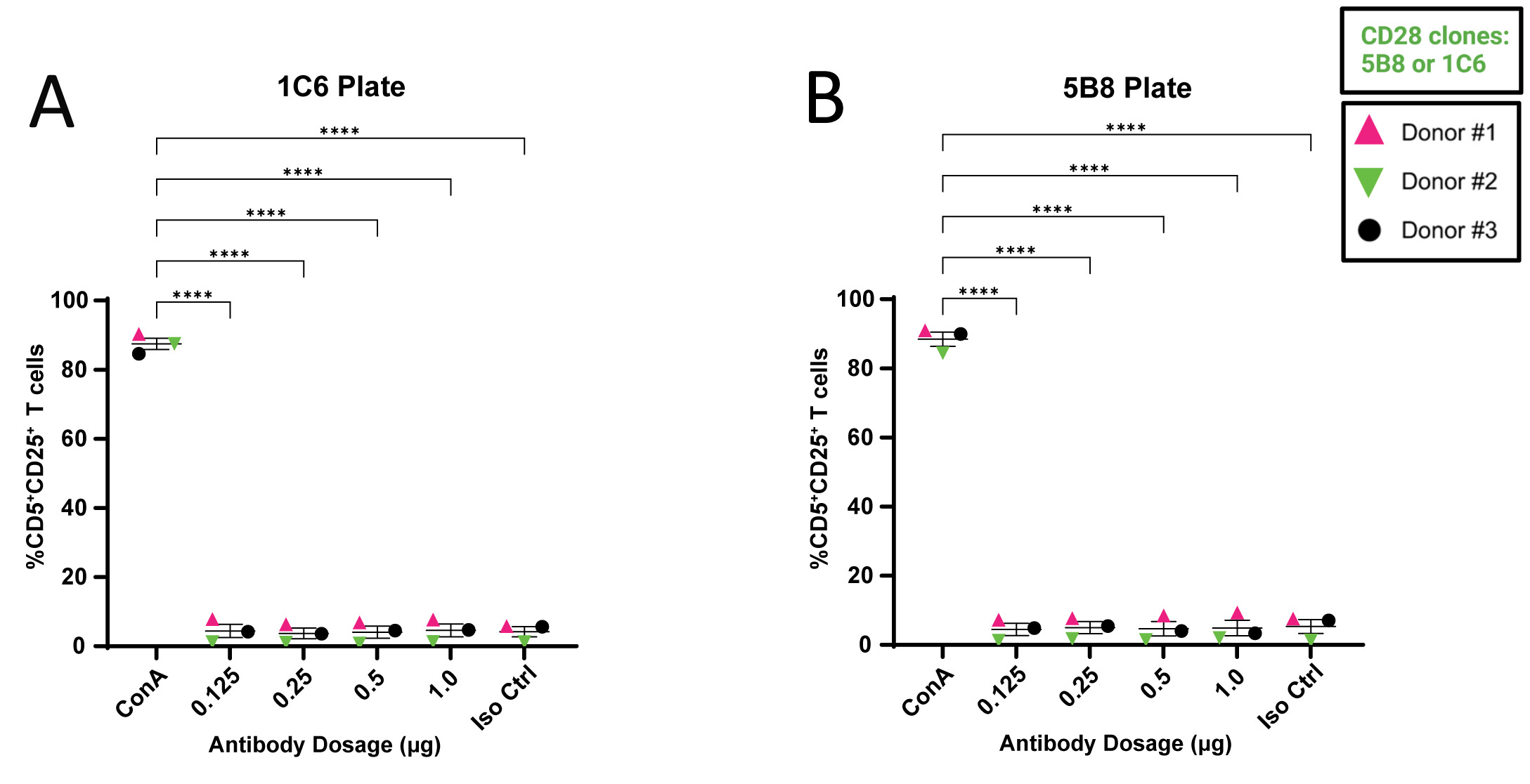

Supplement: S4 Fig — (A-B) Frequency of CD5+ T cell activation in three canine donors as indicated by cell surface expression of CD25. Plate-bound αCD28 antibody (A) 1C6 or (B) 5B8 of different doses was used to determine potential for T cell stimulation when used in the absence of αCD3 antibody. Pairwise statistical analysis was performed by One-way ANOVA with Tukey’s multiple means comparison; **** p < 0.0001. (TIF) [file pone.0324403.s005.tif]

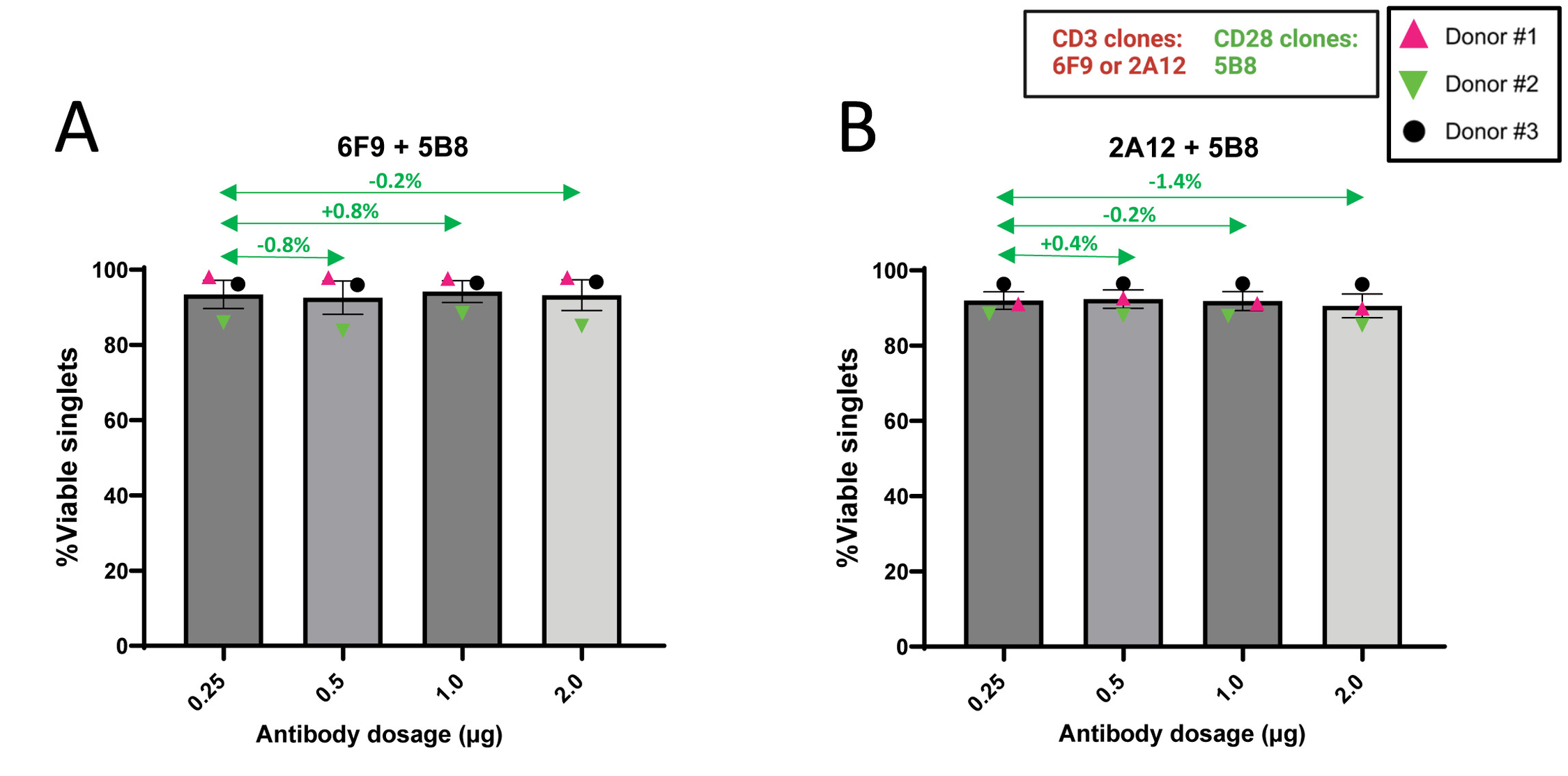

Supplement: S5 Fig — Representation of the effect of dose increase of stimulatory antibody on cell viability. Plate-bound (A) 6F9 + 5B8 and (B) 2A12 + 5B8 were used to stimulate PBMCs from three donors (6F9=CA17.6F9; 2A12=CA17.2A12). On day 3 viability was evaluated by flow cytometry as the frequency of viable singlets. Less than 10% loss in viability was considered acceptable and is indicated by green arrows. Horizontal lines indicate mean values, and error bars represent standard error of the mean. (TIF) [file pone.0324403.s006.tif]

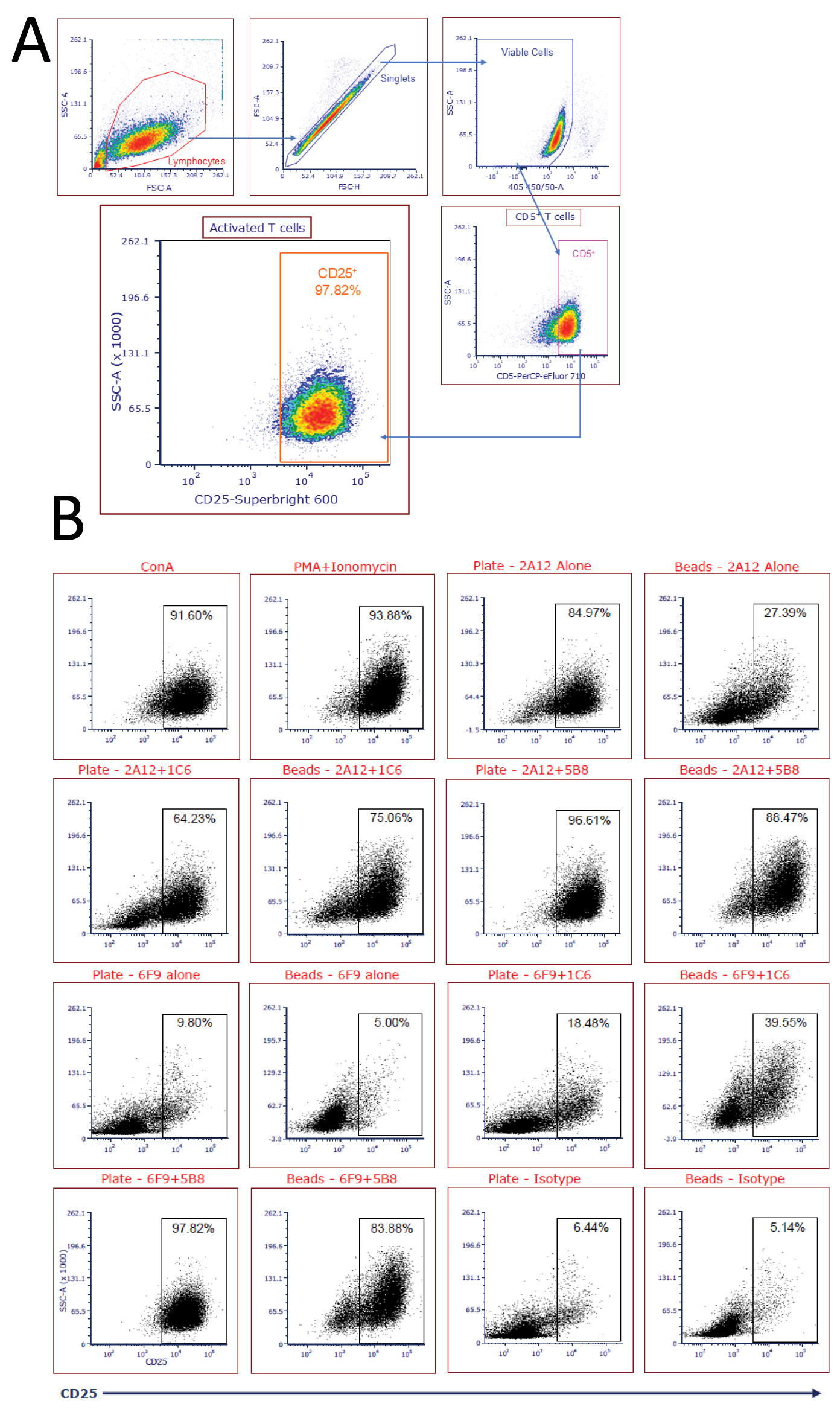

Supplement: S6 Fig — (A) Gating strategy for identifying activated T cells. Lymphocytes are gated in SSC/FSC plot followed by exclusion of doublets. Next, gating on viable cells by exclusion of dead with Live-or-Dye 405/452 viability dye. T cells were identified by gating on CD5+ cells followed by gating on CD25+ cells to identify activated T cells. (B) Representative dot plots for all stimulation strategies are shown (three days post-activation). (TIF) [file pone.0324403.s007.tif]

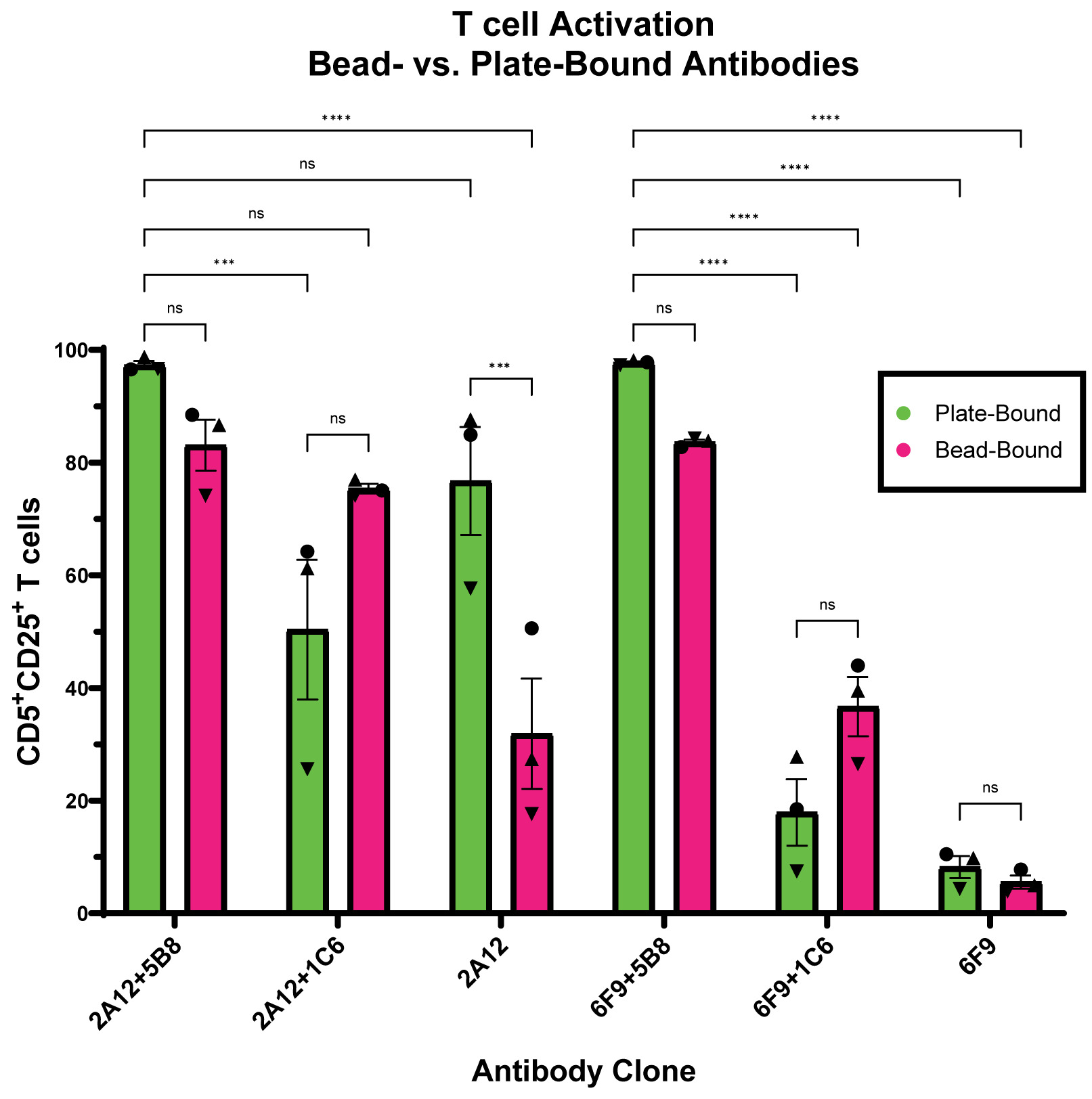

Supplement: S7 Fig — Summary of optimal doses of stimulatory antibodies (6F9=CA17.6F9; 2A12=CA17.2A12) used to activate PBMCs (n = 3); frequency of activated CD5+ T cells was determined by cell surface expression of CD25 after three days. This figure contains the same data from Fig 4 with focused statistical analysis on the effectiveness of antibody clones bound to plates or to beads. Horizontal lines indicate mean values, and error bars represent standard error of the mean. Multiple comparison statistical analysis was performed by Two-way ANOVA with Tukey’s multiple means comparison; *** p < 0.001, **** p < 0.0001, ns not significant. (TIF) [file pone.0324403.s008.tif]

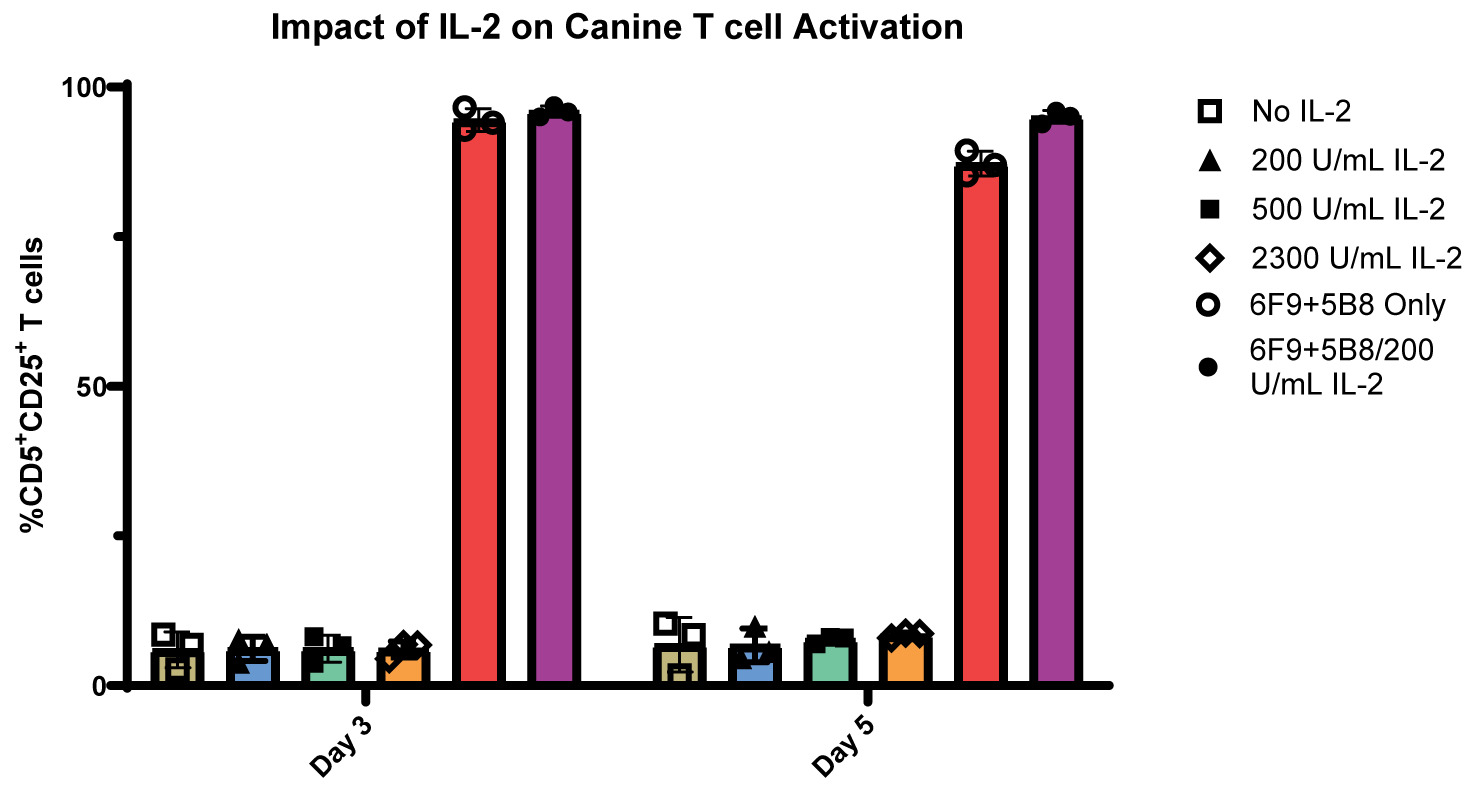

Supplement: S8 Fig — PBMCs from three canine donors were cultured either without stimulation in different concentrations of IL-2 or with stimulation using plate-bound stimulatory antibodies (6F9=CA17.6F9) with or without exogenous IL-2. Unstimulated PBMCs cultured in media with no IL-2 were used as negative controls. Frequency of activated CD5+ T cells was determined by cell surface expression of CD25 at day 3 and 5. Horizontal lines indicate mean values, and error bars represent standard error of the mean. (TIF) [file pone.0324403.s009.tif]

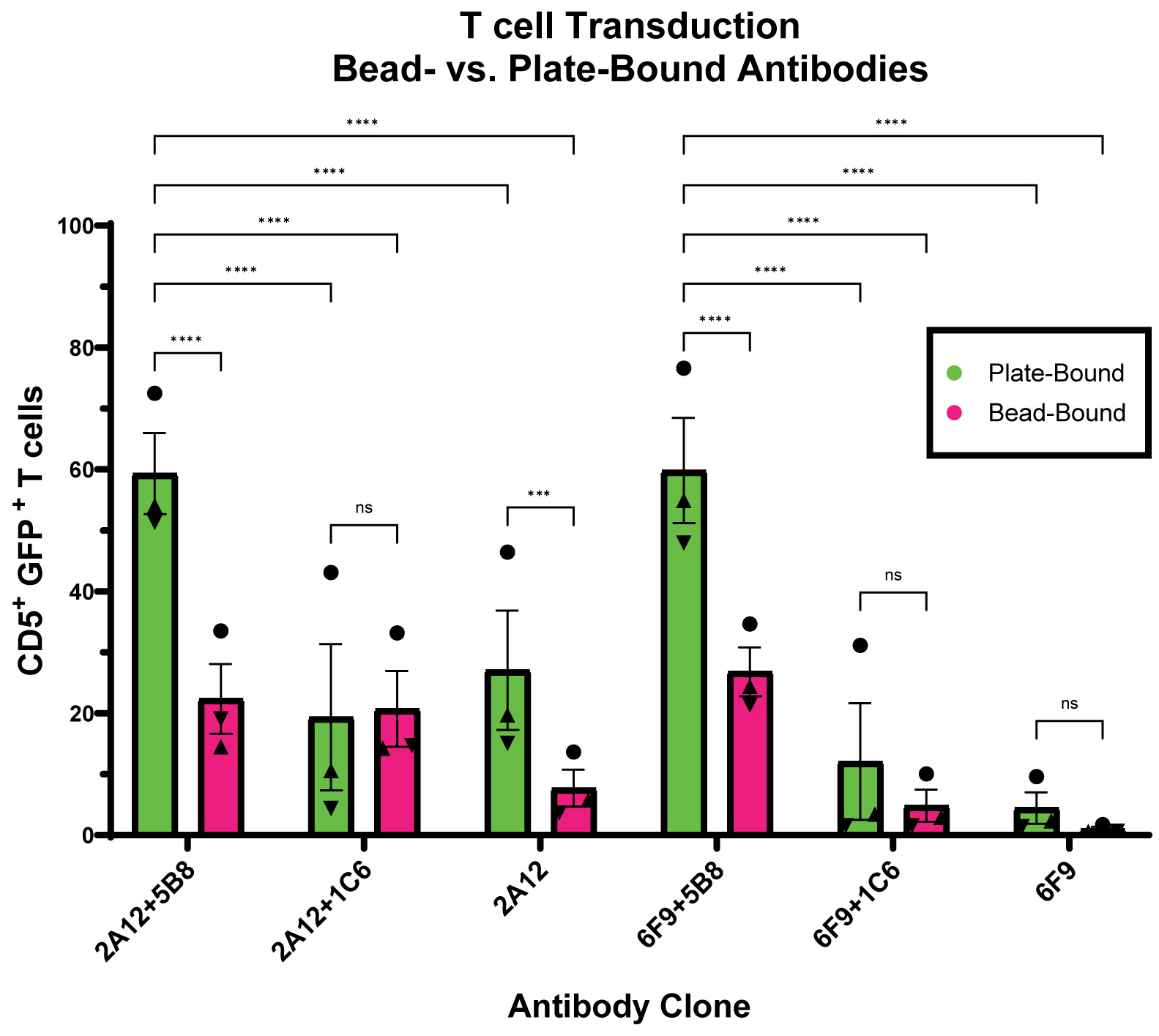

Supplement: S9 Fig — After three days of antibody-induced stimulation (6F9=CA17.6F9; 2A12=CA17.2A12), PBMCs from canine donors (n = 3) were transduced with GFP gamma retrovirus. Frequency of GFP expressing CD5+ T cells was evaluated three days after transduction. This figure contains the same data from Fig 6 with focused statistical analysis on the effectiveness of antibody clones bound to plates or to beads. Horizontal lines indicate mean values, and error bars represent standard error of the mean. Multiple comparison statistical analysis was performed by Two-way ANOVA with Tukey’s multiple means comparison; *** p < 0.001, **** p < 0.0001, ns not significant. (TIF) [file pone.0324403.s010.tif]

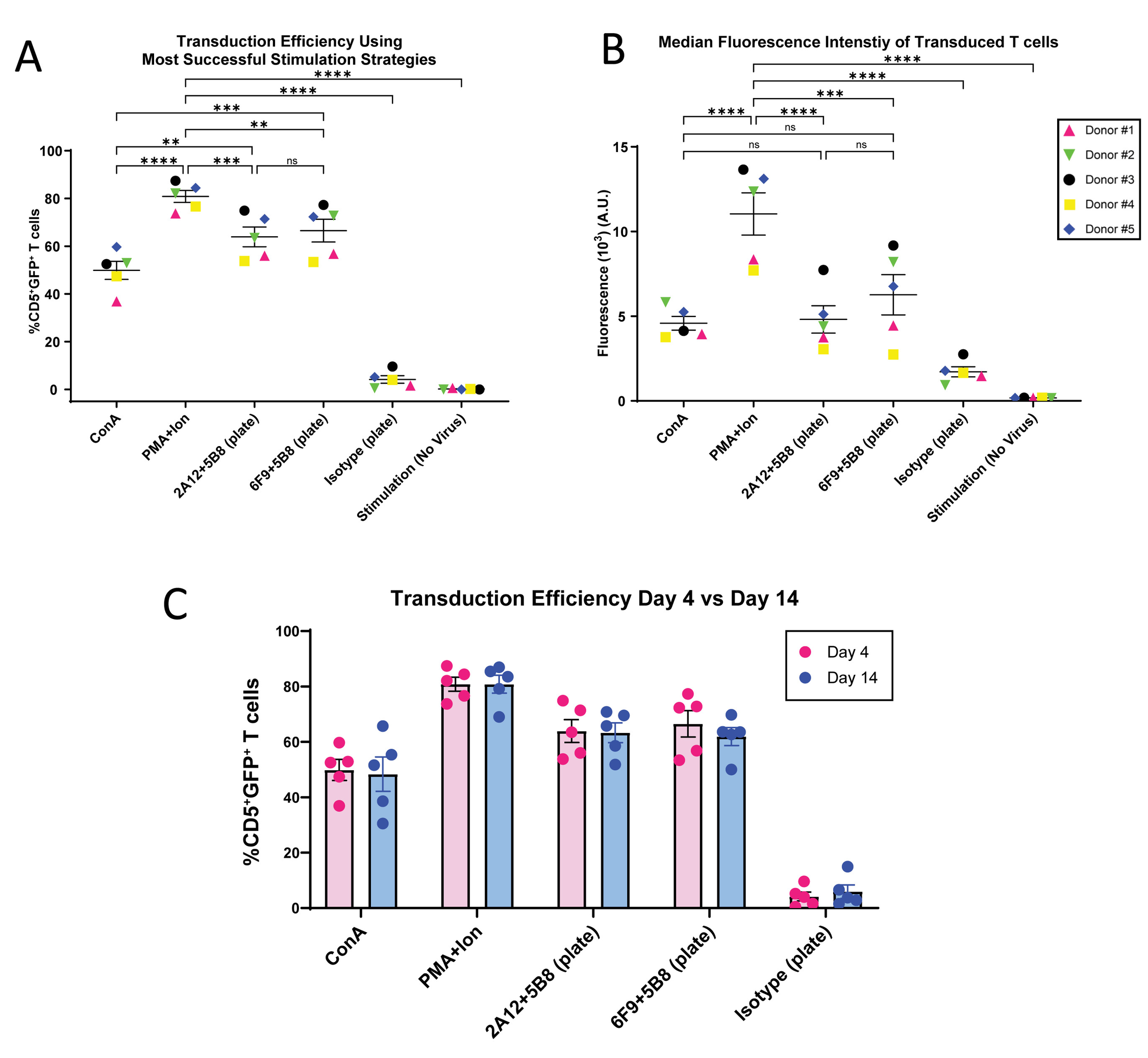

Supplement: S10 Fig — After stimulation with mitogen or plate-bound antibody (6F9=CA17.6F9; 2A12=CA17.2A12), PBMCs (n = 5) were transduced with GFP gamma retrovirus at MOI of 10. (A) Transduction efficiency was determined by the frequency of CD5+ T cells expressing GFP four days after transduction. (B) The intensity of GFP signaling using median fluorescence intensity (MFI) in CD5+GFP+ T cells was used as an indicator of virus particle/cell interaction. (C) To determine persistence of transduced T cells, frequency of GFP+CD5+ T cells was evaluated at day 14 and compared to day 4. Horizontal lines indicate mean values, and error bars represent standard error of the mean. Pairwise statistical analysis was performed by One-way ANOVA with Tukey’s multiple means comparison; ** p < 0.01, *** p < 0.001, **** p < 0.0001, ns no significance. (TIF) [file pone.0324403.s011.tif]

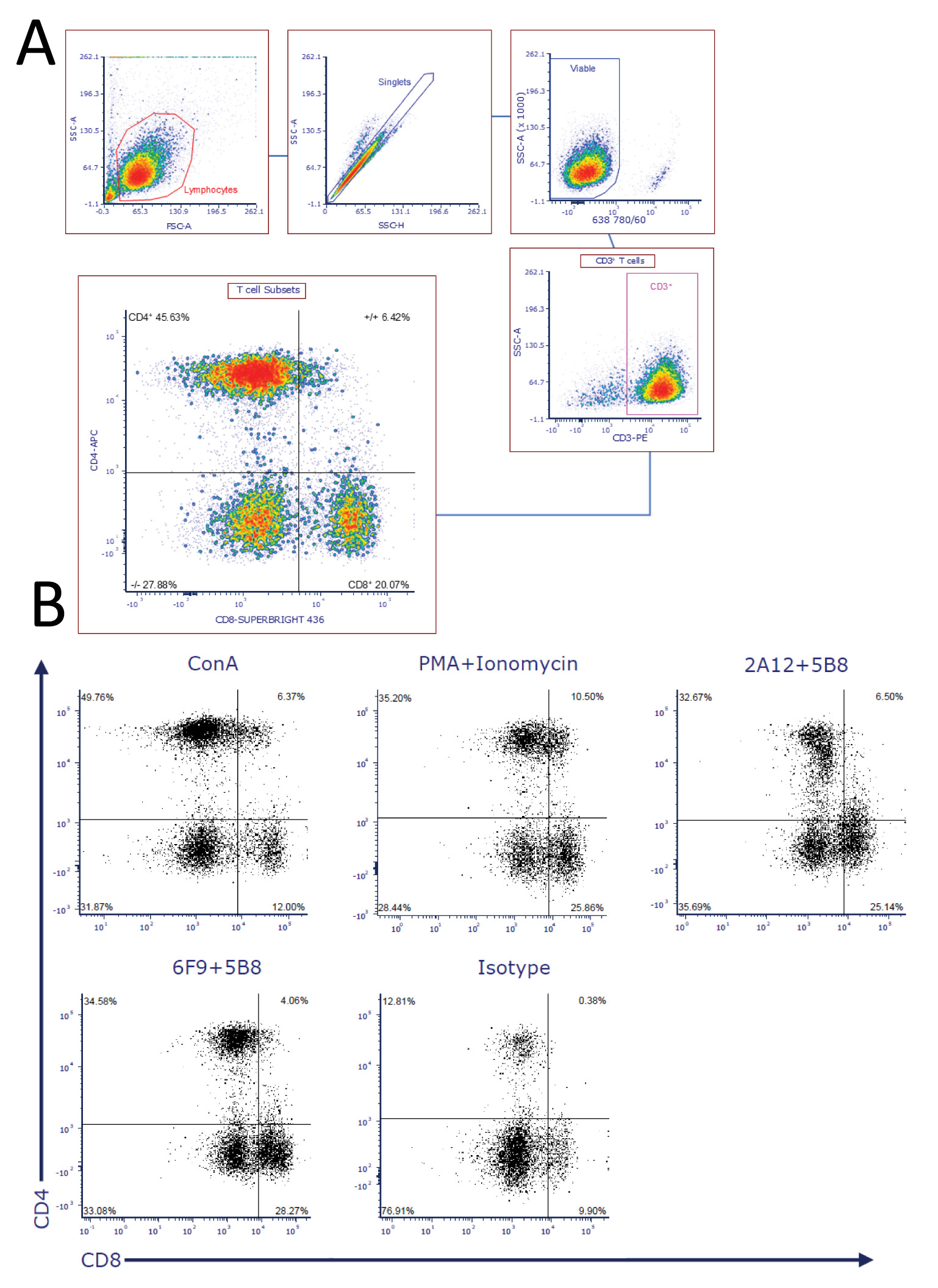

Supplement: S11 Fig — (A) Gating strategy for identifying CD4 and CD8 T cell subsets. Lymphocytes are gated in SSC/FSC plot followed by exclusion of doublets. Next, gating on viable cells by exclusion of dead with LIVE/DEAD Near IR viability dye. T cells were identified by gating on CD3+ cells. Finally, CD4 and CD8 expression were evaluated. Phenotype was determined according to quadrant: Upper left quadrant – CD4+ cells; upper right – double positive cells; lower left – double negative cells; and lower right – CD8+ cells. (B) Representative dot plots for each stimulation strategy are shown (14 days after removal from stimulation). (TIF) [file pone.0324403.s012.tif]

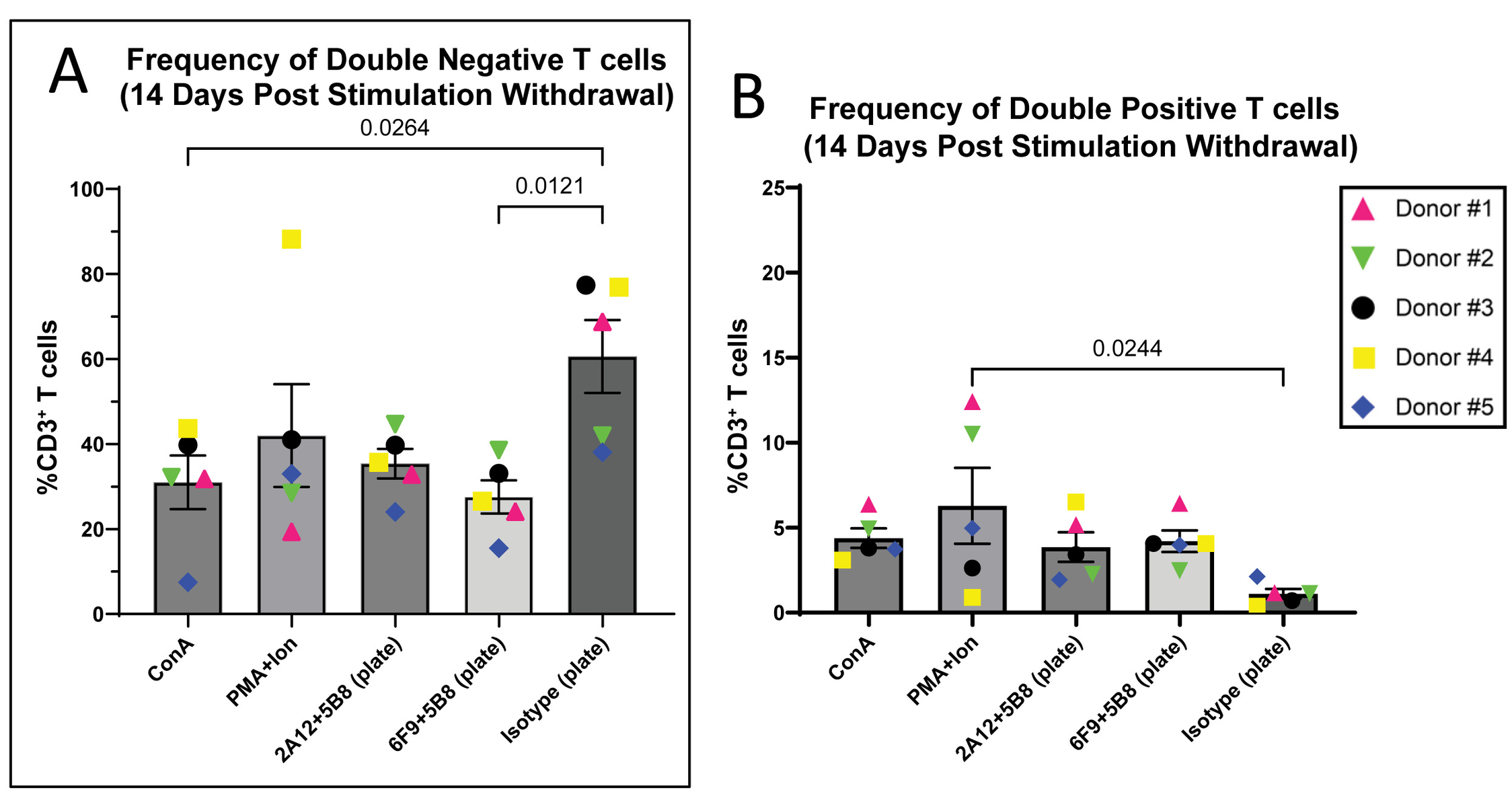

Supplement: S12 Fig — After three days of stimulation with mitogen or plate-bound antibody (6F9=CA17.6F9; 2A12=CA17.2A12), PBMCs (n = 5) were removed from stimulation. Cell surface expression of CD4 and CD8 was evaluated before stimulation (naive), and at 7 and 14 days after removal from stimulation. On day 14, frequency of (A) CD4-CD8- double negative T cells and (B) CD4+CD8+ double positive T cells were characterized. Horizontal lines indicate mean values, and error bars represent standard error of the mean. Pairwise statistical analysis was performed by One-way ANOVA with Tukey’s multiple means comparison; p < 0.05 is considered significant. (TIF) [file pone.0324403.s013.tif]

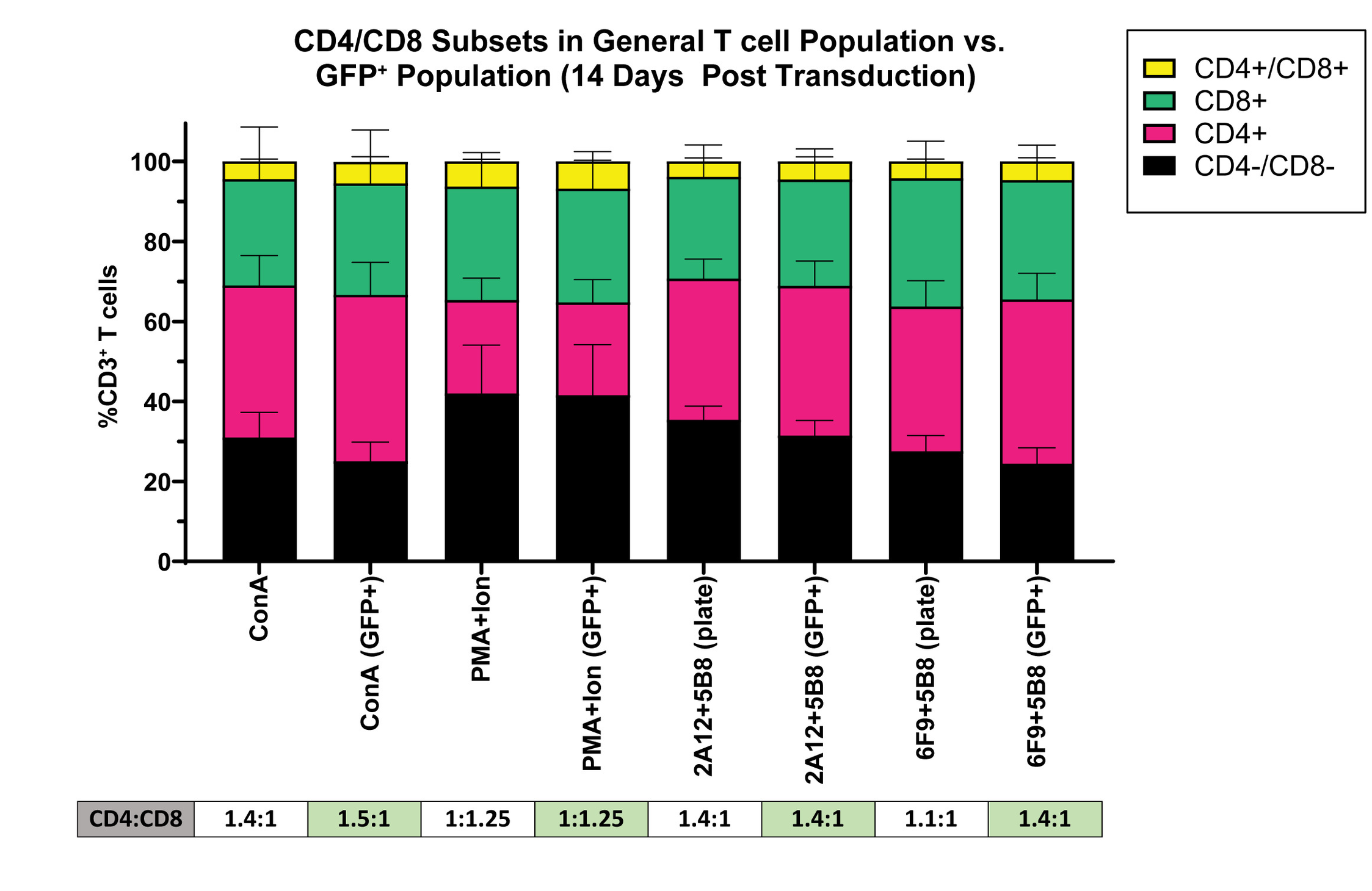

Supplement: S13 Fig — After three days of stimulation with mitogen or plate-bound antibody (6F9=CA17.6F9; 2A12=CA17.2A12), PBMCs (n = 5) were removed from stimulation and transduced with GFP gamma retrovirus. CD4 and CD8 expression was compared on day 14 between the general CD3+ T cell population and transduced GFP+CD3+ T cells. CD4:CD8 ratio was recorded for the general population (white) and the transduced population (green). Horizontal lines indicate mean values, and error bars represent standard error of the mean. (TIF) [file pone.0324403.s014.tif]

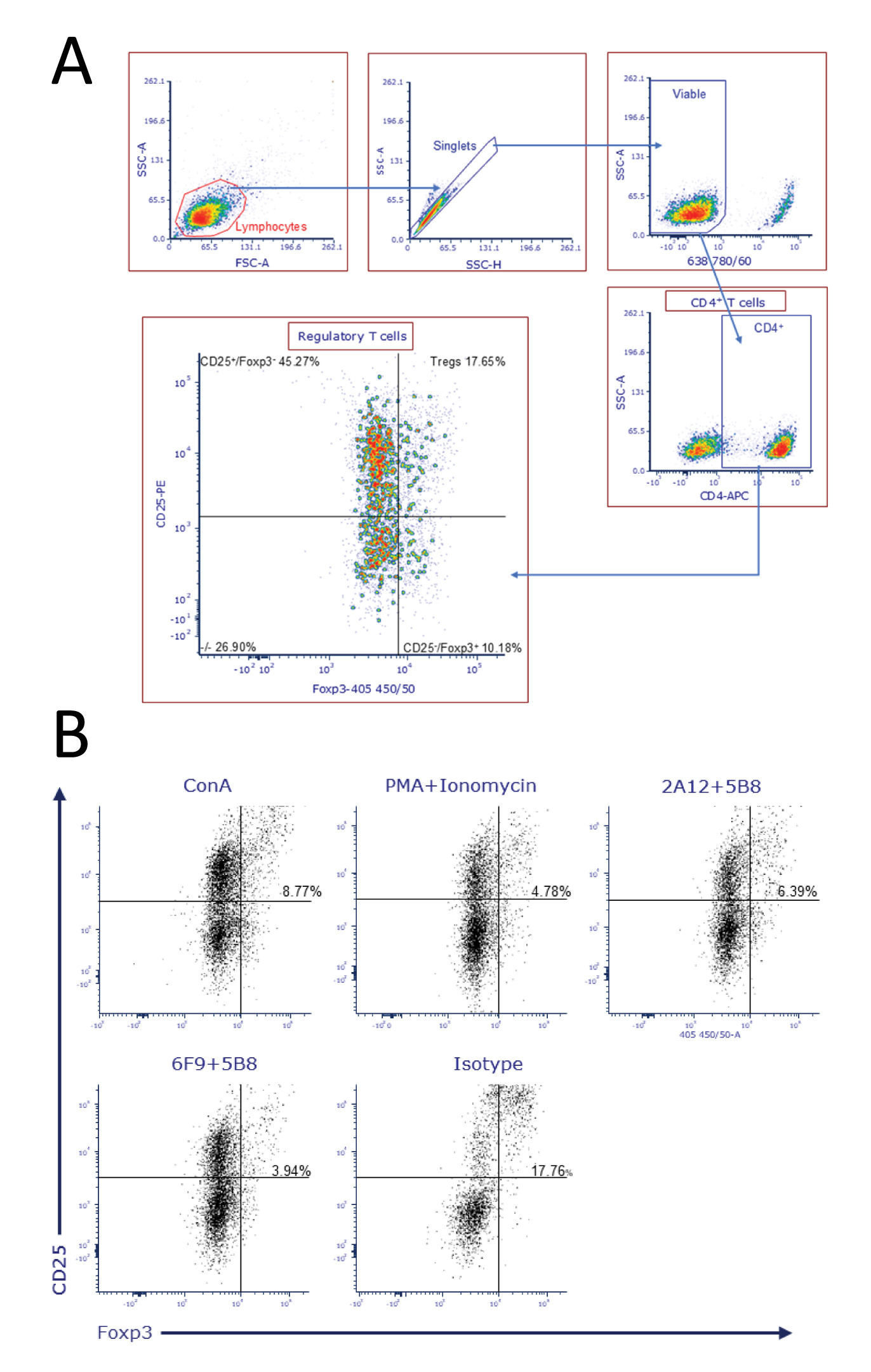

Supplement: S14 Fig — (A) Gating strategy for identifying regulatory T cells. Lymphocytes are gated in SSC/FSC plot followed by exclusion of doublets. Next, gating on viable cells by exclusion of dead with LIVE/DEAD Near IR viability dye. CD4+ T cells were next identified. Finally, CD25 surface expression and intracellular Foxp3 were evaluated. Regulatory T cells were identified as CD25+Foxp3+ cells in the upper right quadrant. (B) Representative dot plots for each stimulation strategy are shown (14 days after removal from stimulation). (TIF) [file pone.0324403.s015.tif]

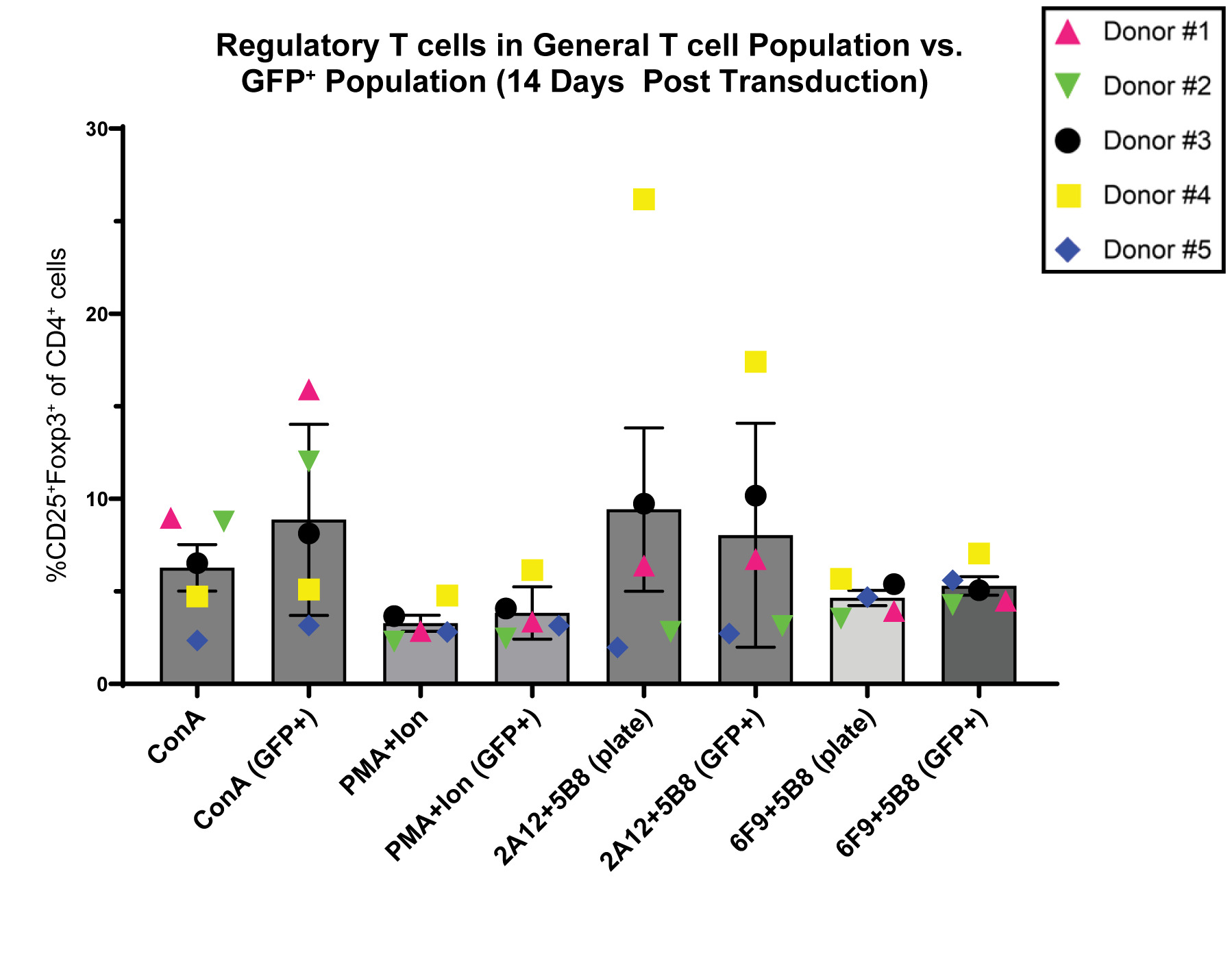

Supplement: S15 Fig — After three days of stimulation with mitogen or plate-bound antibody (6F9=CA17.6F9; 2A12=CA17.2A12), PBMCs (n = 5) were removed from stimulation and transduced with GFP gamma retrovirus. On day 14, the frequency of Tregs was compared between the general CD4+ T cell population and transduced GFP+CD4+ T cells. Horizontal lines indicate mean values, and error bars represent standard error of the mean. (TIF) [file pone.0324403.s016.tif]

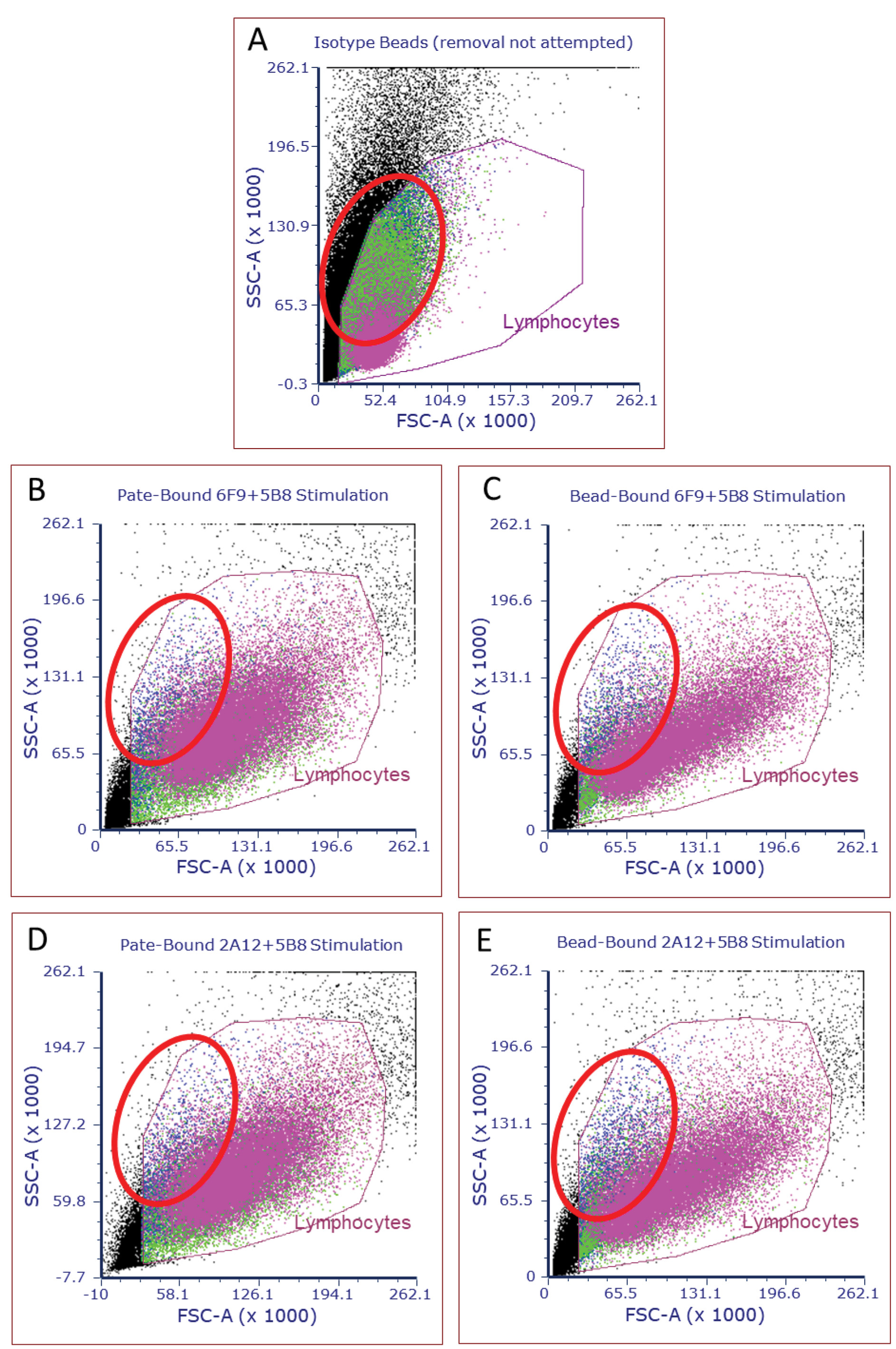

Supplement: S16 Fig — (A) Flow cytometry color dot plot demonstrating bead contamination (green dots within the red circle) in an unstimulated lymphocyte population (pink dots) where the beads were intentionally not removed. (B-C) compares a representative (B) plate-bound 6F9 + 5B8 antibody stimulated (bead free) PBMC population with a representative (C) bead-bound 6F9 + 5B8 antibody stimulated PBMC population from this study. (D-E) compares a representative (D) plate-bound 2A12 + 5B8 antibody stimulated (bead free) PBMC population with a representative (E) bead-bound 2A12 + 5B8 antibody stimulated PBMC population from this study. (TIF) [file pone.0324403.s017.tif]
