# Supplementary material for: Optimizing canine T cell activation, expansion, and transduction
Source: PLoS One. 2025 Sep 11;20(9):e0324403. doi: 10.1371/journal.pone.0324403 (PMC12425254; doi:10.1371/journal.pone.0324403)
Supplement: Attachment — Submitted filename: Davis Resubmission Response.pdf [file pone.0324403.s019.pdf]

Journal Requirements:

When submitting your revision, we need you to address these additional requirements.

1. Please ensure that your manuscript meets PLOS ONE's style requirements, including those for file naming. The PLOS ONE style templates can be found at

https://journals.plos.org/plosone/s/file?id=wjVg/PLOSOne_formatting_sample_main_body.pdf and
https://journals.plos.org/plosone/s/file?id=ba62/PLOSOne_formatting_sample_title_authors_affiliations.pdf

We noted several errors where “Fig” should read “Figs” and all were edited.
“address” was removed in “Email address”
Please let us know what other formatting errors exist.

2. We note that the grant information you provided in the ‘Funding Information’ and ‘Financial Disclosure’ sections do not match.

When you resubmit, please ensure that you provide the correct grant numbers for the awards you received for your study in the ‘Funding Information’ section.

The Funding Information section was revised.

3. Thank you for stating in your Funding Statement:

[This work was supported by the National Institutes of Health through Grant Award R37-CA260223 and R33-CA281875 from the NCI, seed funding from the Comparative Medicine Institute and the Lineberger Cancer Center and by start-up funds provided by the University of North Carolina at Chapel Hill, North Carolina State University at Raleigh, and the Lineberger Cancer Center.].
Please provide an amended statement that declares *all* the funding or sources of support (whether external or internal to your organization) received during this study, as detailed online in our guide for authors at http://journals.plos.org/plosone/s/submit-now. Please also include the statement “There was no additional external funding received for this study.” in your updated Funding Statement.
Please include your amended Funding Statement within your cover letter. We will change the online submission form on your behalf.

The cover letter was amended to include the following:

> “This work was supported by the National Institutes of Health through Grant Award R37-CA260223 and R33-CA281875 from the National Cancer Institute, a seed grant from the Comparative Medicine Institute, a UNC Lineberger Developmental Grant, an RISF grant from NC State and by start-up funds provided by the University of North Carolina at Chapel Hill, North Carolina State University at Raleigh, and the Lineberger Cancer Center. T.D. was in part funded by a Genetics & Genomics Scholars Fellowship and by an administrative supplement to the R37CA260223. There was no additional external funding received for this study.

4. Thank you for stating the following in the Acknowledgments Section of your manuscript:

[This work was supported by the National Institutes of Health through Grant Award R37-CA260223 and R33-CA281875 from the NCI, seed funding from the Comparative Medicine Institute and the Lineberger Cancer Center and by start-up funds provided by the University of North Carolina at Chapel Hill, North Carolina State University at Raleigh, and the Lineberger Cancer Center. We are grateful to Peter Moore (UC Davis) for gifting the αCD3 antibody (clone CA17.6F9); Brian Hayes (Fred Hutchinson Cancer Center) for gifting the 5B8 hybridoma cell line; Gianpietro Dotti (UNC) for gifting the GFP gamma retrovirus and Drs. Christopher DeRenzo, Dr. Stephen Gottschalk, and Carla O'Reilly (St. Jude Children's Research Hospital for their time and support in discussing canine T cell transduction. Additionally, we thank NCSU Laboratory Animal Resources veterinary medical team and North American Veterinary Blood Bank for their collection and donation of canine whole blood and David Rose (NCSU) for his support in flow cytometry data collection.]
We note that you have provided funding information that is currently declared in your Funding Statement. However, funding information should not appear in the Acknowledgments section or other areas of your manuscript. We will only publish funding information present in the Funding Statement section of the online submission form.
Please remove any funding-related text from the manuscript and let us know how you would like to update your Funding Statement. Currently, your Funding Statement reads as follows:

[This work was supported by the National Institutes of Health through Grant Award R37-CA260223 and R33-CA281875 from the NCI, seed funding from the Comparative Medicine Institute and the Lineberger Cancer Center and by start-up funds provided by the University of North Carolina at Chapel Hill, North Carolina State University at Raleigh, and the Lineberger Cancer Center.]

Please include your amended statements within your cover letter; we will change the online submission form on your behalf.

Statements related to funding were removed from the manuscript and the updated Financial Statement was added to the cover letter as described above.

5. Thank you for stating the following in the Competing Interests section:

[The authors declare the following financial interests/personal relationships which may be considered as potential competing interests: Yevgeny Brudno has patents related to CAR T cell production and therapy pending to NC State. Yevgeny Brudno is the scientific founder of Persistence Therapeutics, which seeks novel methods for CAR T cells therapy. None of the other authors have known competing financial interests or personal relationships that could have appeared to influence the work reported in this paper].

Please confirm that this does not alter your adherence to all PLOS ONE policies on sharing data and materials, by including the following statement: "This does not alter our adherence to PLOS ONE policies on sharing data and materials." (as detailed online in our guide for authors http://journals.plos.org/plosone/s/competing-interests). If there are restrictions on sharing of data and/or materials, please state these. Please note that we cannot proceed with consideration of your article until this information has been declared.

The Competing Interests Section was removed from the manuscript. The cover letter was amended to include the following in the **Competing Interests Statement**:

> "The authors declare the following financial interests/personal relationships which may be considered as potential competing interests: Yevgeny Brudno has licensed patents

related to CAR T cell production and therapy pending to NC State and UNC. Yevgeny Brudno is the scientific founder of Persistence Therapeutics, which seeks novel methods for CAR T cells therapy. None of the other authors have known competing financial interests or personal relationships that could have appeared to influence the work reported in this paper. These conflicts do not alter our adherence to PLOS ONE policies on sharing data and materials."

Please include your updated Competing Interests statement in your cover letter; we will change the online submission form on your behalf.

If the reviewer comments include a recommendation to cite specific previously published works, please review and evaluate these publications to determine whether they are relevant and should be cited. There is no requirement to cite these works unless the editor has indicated otherwise.

There were no requests to cite specific works.

Response to Reviewers

We sincerely thank all four reviewers for their thorough and constructive evaluation of our manuscript. We are pleased that all reviewers unanimously agreed that our work is technically sound with data that supports our conclusions, that our statistical analysis was performed appropriately and rigorously, that we have made all underlying data fully available in accordance with PLOS policies, and that our manuscript is presented in an intelligible fashion with clear, standard English. This consensus among the reviewers validates our systematic approach to optimizing canine T cell activation, expansion, and transduction strategies, and we appreciate their recognition of the scientific rigor and clarity of our work.

Reviewer #1: This manuscript presents a systematic and detailed study on the optimization of canine T cell activation, expansion, and lentiviral transduction. Given the emerging role of dogs as translational models for human cancer immunotherapy, the work addresses a timely and important challenge: the lack of standardized and effective T cell activation protocols in canines. The authors evaluate different αCD3 and αCD28 antibody clones using bead- and plate-bound formats, compare their performance to mitogens, and assess IL-2's role in canine T cell biology. They identify plate-bound αCD3 clone 6F9 or 2A12 with αCD28 clone 5B8 co-stimulation as the most effective strategy for activation and expansion, providing a valuable framework for canine adoptive T cell therapy development.

The data are generally well-conceived, and the experiments are thorough and replicable.

We appreciate the reviewer's kind words and the time the reviewer spent to read and review the manuscript.

However, the manuscript would benefit significantly from improved organization, more robust statistical treatment, and a deeper discussion of clinical and translational implications.
1.The introduction and abstract lack a clearly stated central hypothesis or specific research aims.
Recommendation: State explicitly in the introduction whether the goal is to identify an optimal T cell activation protocol, compare bead vs. plate systems, or evaluate mitogens in the context of gene therapy. This framing should guide the manuscript structure.

This would indeed be helpful for the reader. We have added to the final paragraph of the *Introduction* a clearly stated research aim as follows:

> "The central aim of our work was to identify and optimize a stimulation protocol yielding robust canine T cell activation for optimized cell transduction and expansion towards benefiting canine patient T cell immunotherapy."

2.The discussion would benefit from elaboration on how these findings might be used in:

Preclinical trials in dogs, Companion animal cancer therapy, Bridging to human T cell engineering.

Recommendation: Add a focused paragraph in the discussion on future directions and clinical translation, including whether GMP-compliant reagents (or alternatives) for 5B8/6F9 are available or needed.

Per your recommendation, we have added the following paragraph to the end of the DISCUSSION section:

> "While additional work remains to achieve GMP compliance for the 6F9 and 5B8 antibodies, these findings lay critical groundwork for preclinical and clinical trials in dogs with cancers. Such studies will enable comprehensive assessment of CAR T cell safety, expansion, and therapeutic function in vivo. The protocols described here could enhance both the feasibility and accessibility of CAR T cell therapy in veterinary onccology. Importantly, canine studies are increasingly valuable translational bridges to human immunotherapy. Compared to murine studies, dogs with spontaneous disease provide a more immunologically relevant and heterogeneous model. This comparative oncology approach will not only strengthen the translational research pipeline but also will accelerate development of next-generation human therapies

3.CD25 is a useful early activation marker but provides limited insight into T cell functionality. Recommendation: Include additional activation or functional readouts such as CD69, cytokine production (e.g., IFN-γ, IL-2), or cytotoxic markers to better validate the functional status of activated cells.

We appreciate the reviewer's point regarding the usefulness of evaluating CD69. While evaluating CD69 would have been ideal - and it is a routine part of our human and murine T cell studies - antibodies for canine CD69 are not currently commercially (or otherwise) available, making CD69 assessment not possible. Although others have used detection of CD69 mRNA (Zhe et al.), we do not feel that mRNA is adequately reflective of CD69 surface marker presentation. To highlight this important point, we have made the following changes to the METHODS section under "Mitogen preparation and use for activation assay" heading for clarification:

> "After three days, CD25 expression was evaluated by flow cytometry to determine activation frequency. Evaluating CD69 expression was considered, but anti-canine CD69 antibodies are not currently commercially available and reliable cross-reactive CD69-specific monoclonal antibodies used in other species have not been identified[20]"

We also agree that functionality is a critical piece as we move to clinical trials. However, we feel that cytokine production and cytotoxicity would best be evaluated (and we hope you agree) in the context of a CAR-expressive T cell. This study's primary objective was to determine the best stimulations strategy for canine activation, which is a necessary first step to CAR expression. Robust expansion is necessary for function, but is critical for gamma retrovirus transduction. As such, we used GFP as a marker of transduction, not CAR. We are actively working to optimize production of antigen-specific canine CAR T cells using the stimulation strategies identified in this manuscript. We hope to soon report optimized virus and CAR constructs for very robust CAR T cell production.

We have modified the paragraph on limitations in the DISCUSSION to specify CAR T cell use and included cytokine analysis as a measure of functionality as follows:

> "This study has several limitations. First, all transduction experiments utilized GFP gamma retrovirus with GFP serving as the marker for successful transduction. However, the metabolic demands of GFP production differ from those required for the much larger CAR protein, and cell surface CAR expression may not correlate with GFP expression. Second, while transduction efficiency and T cell phenotype serve as predictors for therapeutic potential, they do not necessarily translate to superior functionality. Future work will explore how the optimized stimulation identified here can be harnessed to produce tumor antigen-specific CAR T cells, including functional assays to assess target cell killing, cytokine production, T cell exhaustion, and memory differentiation [88–90]."

4.The manuscript concludes that 5B8 is superior to 1C6, but does not explore why. Similarly, the antagonistic effect of plate-bound 1C6 is described but not investigated.

Recommendation: Consider brief experiments or discussion on possible mechanisms (e.g., differences in epitope binding, receptor internalization, or Fc-mediated effects).

The reviewer brings up an excellent point. We were also surprised by this result. We believe that T cell responses to 1C6 are dictated by the presentation of the antibody, but beyond this we don't have a coherent hypothesis. We considered the specific differences of a beads surface to a plate's surface and offered the following as an explanation for the difference in T cell response:

> "In contrast to beads, where 1C6 co-stimulation enhanced activation, plate-bound 1C6 failed to improve activation over αCD3 antibodies alone, and in fact appeared to be inhibitory with 2A12. While the contradictory behavior of 1C6 antibody was perplexing, we propose that differences in antibody-substrate interaction and the subsequent presentation to T cells provide the most plausible explanation. High affinity antibody binding to MACSiBeads results in a substrate with predictable antibody orientation, high surface density, spherical topology, and dynamic mobility. Conversely, passive adsorption to polystyrene plates results in variable antibody orientation [33] and comparatively lower surface density [48] on a flat and rigid substrate. These contrasting conditions likely alter crosslinking behavior, create differential steric hindrance effects, and modify T cell receptor engagement, collectively modulating 1C6 downstream intracellular signaling"

Regarding a possible mechanism for 5B8 superiority of 1C6, we added:

> "As with beads, 5B8 outperformed 1C6 in the plate-bound format. Since early reports suggest both clones bind CD28 with similar efficiency [36], the difference in activity is likely due to variations in the binding site or binding affinity, resulting in enhanced intracellular signaling with 5B8."

Reviewer #2: Treyvon W. Davis et al. systematically evaluated various stimulation strategies to optimize activation, expansion, and retroviral transduction of canine T cells for immunotherapeutic applications. The study demonstrates that plate-bound antibodies significantly outperform bead-bound antibodies, and that stimulation with the αCD3 clone CA17.6F9 combined with αCD28 clone 5B8—or with the mitogen PMA plus ionomycin—yields superior T cell activation, expansion, transduction efficiency, and phenotypic profiles. These findings offer a valuable framework for advancing canine CAR T cell therapies, with implications for both veterinary medicine and translational human immunotherapy research. I have the following comments and questions:

We appreciate the reviewer's kind words and the time the reviewer spent to read and review the manuscript.

1. I recommend that the authors provide a more detailed background on the two αCD3 clones (CA17.2A12 and CA17.6F9) and the two αCD28 clones (1C6 and 5B8) in the Introduction or Methods section. This would help readers better understand the rationale behind selecting and comparing these antibody combinations.

Thank you for the opportunity to clarify. The rationale for selecting these specific antibodies was the goal of cataloguing the stimulatory effects of EVERY available agonistic antibody described in canine literature (which was limited to these four clones).

We clarified this in the final paragraph of the introduction as follows:

> "The central aim of our work was to identify and optimize a stimulation protocol yielding robust canine T cell activation for optimized cell transduction and expansion towards benefiting patient canine T cell immunotherapy. As such, we sought to directly compare the efficacy of all possible combinations of stimulatory antibodies for canine CD3 and CD28 described in the literature."

Additionally, we provided the following background information for the antibody clones in the INTRODUCTION:

> "2A12 was first reported in 1993 by Moore and Rossitto [35] to aid in the diagnosis of T cell neoplasia. The origin of 6F9 is less well documented, but it was supplied by Moore's group and used by Graves et al. in 2011 [36], where it was used in combination with the newly developed αCD28 antibodies 5B8 or 1C6 to promote T cell expansion. In that context, expansion was enhanced by 5B8 co-stimulation, while it was inhibited by 1C6. As a result, 1C6 was initially characterized as antagonistic, but it has since been used by multiple groups to successfully stimulate canine T cells [16,20,34,37]."

2. The study relies on CD25 expression to assess T cell activation. The authors should clarify why CD25 was chosen over the more commonly used early activation marker CD69, and whether any comparative data were available.

While evaluating CD69 would have been ideal - and it is a routine part of our human and murine T cell studies - antibodies for canine CD69 are not commercially (or otherwise) available. As a result, we were unable to assess CD69 expression. Although others have used detection of CD69 mRNA (Zhe et al.), we do not feel that mRNA is adequately reflective of CD69 surface marker presentation. To highlight this important point, we have made the following changes to the METHODS section under "Mitogen preparation and use for activation assay" for clarification:

> "After three days, CD25 expression was evaluated by flow cytometry to determine activation frequency. Evaluating CD69 expression was considered, but anti-canine CD69 antibodies are not currently commercially available and reliable cross-reactive CD69-specific monoclonal antibodies used in other species have not been identified[20]."

3. The abbreviations "2A12" and "6F9" are first introduced at line 379. For clarity and consistency, I suggest defining these abbreviations earlier in the manuscript, ideally when the antibodies are first mentioned.

At first mention, the abbreviations "2A12" and "6F9" were added in the introduction as follows:

> "Two αCD3 antibody clones, CA17.2A12 (2A12) and CA17.6F9 (6F9) and two αCD28 antibody clones, 1C6 and 5B8, have been described..."

4. For improved readability, I recommend moving all figure legends to the end of the manuscript, rather than interspersing them within the main text.

While we agree with the reviewer, PLOS One guidelines mandate the format or our manuscript. From the PLOS One submission guidelines website (https://journals.plos.org/plosone/s/submission-guidelines):

> "Figure captions are inserted immediately after the first paragraph in which the figure is cited. Figure files are uploaded separately."

5. The study exclusively used fresh or thawed primary canine PBMCs rather than established T cell lines. I encourage the authors to explicitly state this in the Discussion section and to comment on the benefits and limitations of using primary cells—particularly in terms of reproducibility, cell heterogeneity, and functional variability.

We appreciate the point. As requested, we also added the following to this portion of the conclusion:

> "All experiments in this report were performed using cryopreserved canine PBMCs from healthy canine donors (S1 Table). Primary PBMCs were selected over established canine T cell lines because robust stimulation—a critical element of this research—would not be physiologically relevant in immortalized cell lines. Additionally, since CAR T cell therapies almost always use autologous primary cells, we reasoned that using primary cells from genetically diverse donors strengthened the therapeutic relevance and applicability of findings to the broader veterinary population. Future studies including more breed variety and clinically affected dogs will further enhance translational relevance. Future work could also utilize isolated T cells, which would likely reduce T cell transduction and expansion variability and minimize differences attributable to the inherent cellular heterogeneity of PBMCs."

6. In Figure S8, I recommend showing individual data points to provide better visual representation of biological variability.

In light of your recommendation and the recommendation of another reviewer, we decided that instead of revising S8 which was extremely similar to Fig. 6, we completely replaced it with an alternative analysis that provides the same information while providing a direct comparison of antibody clone effectiveness in the context of bead vs. plate presentation using Two-way ANOVA statistical analysis. We were careful to include individual data points per your recommendation. See figure and caption below. NOTE: S8 is now S9.

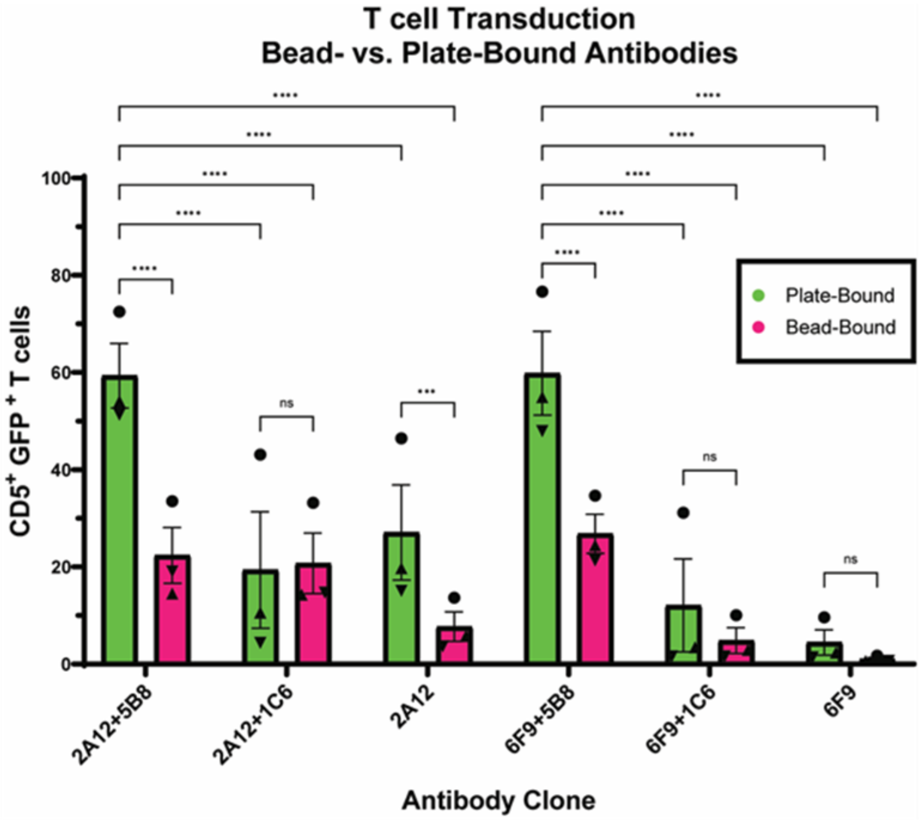

S9 Fig. Plate-bound αCD3 with 5B8 co-stimulation results in better transduction efficiency than bead-bound antibody. After three days of antibody-induced stimulation (6F9=CA17.6F9; 2A12=CA17.2A12), PBMCs from canine donors (n=3) were transduced with GFP gamma retrovirus. Frequency of GFP expressing $CD5^{+}$ T cells was evaluated three days after transduction. This figure contains the same data from Fig. 6 with focused statistical analysis on the effectiveness of antibody clones bound to plates or to beads. Horizontal lines indicate mean values, and error bars represent standard error of the mean. Multiple comparison statistical analysis was performed by Two-way ANOVA with Tukey's multiple means comparison; *** $p<0.001$, **** $p<0.0001$, ns not significant

Reviewer #3: In this manuscript, the authors present an extensive and well-executed screening of optimal conditions for canine T cell transduction. Their study includes twelve antibody stimulation strategies, two previously untested mitogens, multiple αCD3/αCD28 combinations, and a comparison of the two most widely used antibody presentation formats. The methodology, results, figure legends, and discussion are all presented with a high degree of clarity, logic, and scientific rigor. Overall, this work makes a valuable contribution to the field.

We appreciate the reviewer's kind words and the time the reviewer spent to read and review the manuscript.

There are, however, a few formatting and presentation issues that should be addressed to further improve the quality and clarity of the manuscript. My comments are as follows:

Thank you for catching all these formatting errors and errors in the figure legends!

1) Line 133. The formatting of "x" in the phrase preceding "DPBS" appears incorrect. Please review and standardize the formatting of this term throughout the manuscript.
The formatting was changed to "1x Dulbecco's phosphate-buffered saline (DPBS)" in the manuscript. Additionally, 1X was removed from DPBS throughout the manuscript as it is redundant

2) Line 135. The format used to state centrifugation speed is incorrect. Please check all instances where centrifugation speed is stated and revise for consistency.

Formatting was changed to "25 mL of dilute blood was added to the Lymphoprep layer and centrifuged for 40 minutes at 400 x g with lowest acceleration setting and no brake."

3) Line 349. There's an unusual blank space between lines 349 and 350.

The unusual blank space was deleted

4) Fig. 1. In the legend of Fig. 1, the authors state that * $p < 0.05$ is used, but such a statistical significance marker is not shown in panels A or C.

* $p < 0.05$ was removed from the figure legend

5) Fig. 4. Statistics. While one-way ANOVA with Tukey's multiple comparisons is statistically valid for multiple group comparisons, a Two-way ANOVA accounting for antibody clone and stimulation method would better reflect the factorial design of this experiment.

While we feel that the current statistical analysis is important and the best way to make comparisons between all stimulation strategies used concurrently in this case, we also agree that a Two-way ANOVA would add valuable statistical insights in the direct comparison of antibody clone effectiveness in the context of bead vs. plate presentation. In light of your recommendation, we have made a supplemental figure to accompany Fig. 4 that specifically analyzes this question using Two-way ANOVA statistical analysis. See new figure and caption on new S7 Fig:

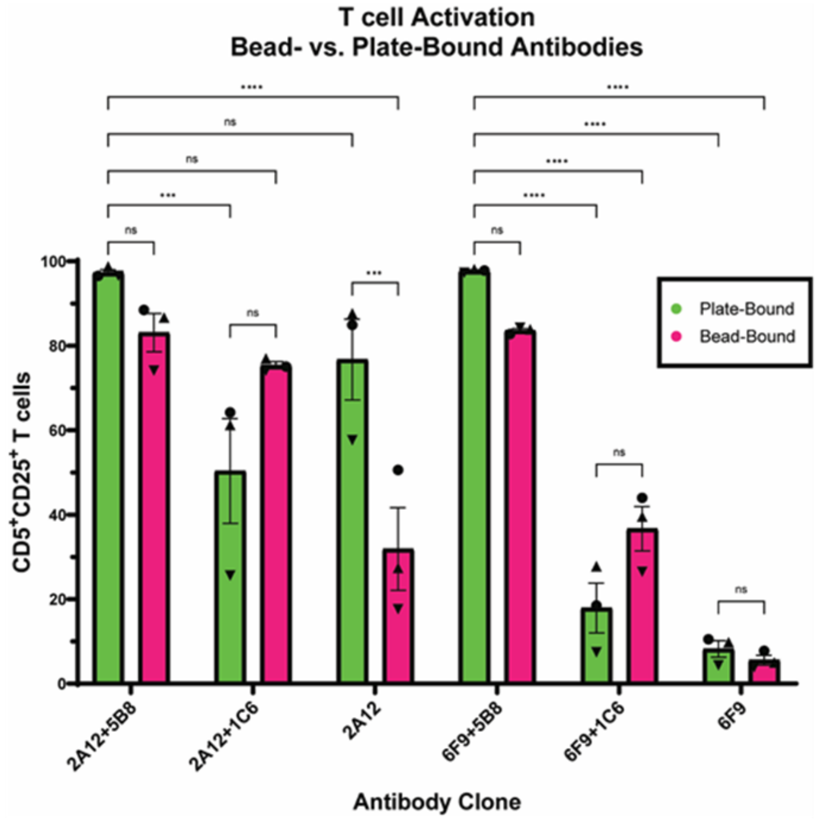

S7 Fig. Plate-bound αCD3 with 5B8 co-stimulation results in better activation than bead-bound antibody. Summary of optimal doses of stimulatory antibodies (6F9=CA17.6F9; 2A12=CA17.2A12) used to activate PBMCs (n=3); frequency of activated $CD5^{-}$ T cells was determined by cell surface expression of CD25 after three days. This figure contains the same data from Fig. 4 with focused statistical analysis on the effectiveness of antibody clones bound to plates or to beads. Horizontal lines indicate mean values, and error bars represent standard error of the mean. Multiple comparison statistical analysis was performed by Two-way ANOVA with Tukey's multiple means comparison; *** $p<0.001$, **** $p<0.0001$, ns not significant

6) Fig. 6. The same statistical analysis approach used in Fig. 4 should also be applied to Fig. 6. This recommendation also applies to Fig. S8A and S8B.

We again feel that the current statistical analysis is important and the best way to make comparisons between all stimulation strategies used concurrently in this case, but we agree that a Two-way ANOVA would add valuable statistical insights in the direct comparison of antibody clone effectiveness in the context of bead vs. plate presentation. Instead of revising S8 which was extremely similar to Fig. 6, we completely replaced it with an alternative analysis similar to the new S7 Fig above. We are grateful for your suggestion as with your guidance we have made a better figure that gives the same information as the previous figure while providing new relevant statistical information using Two-way ANOVA with Tukey's multiple comparisons. See figure and caption below. NOTE: S8 is now S9.

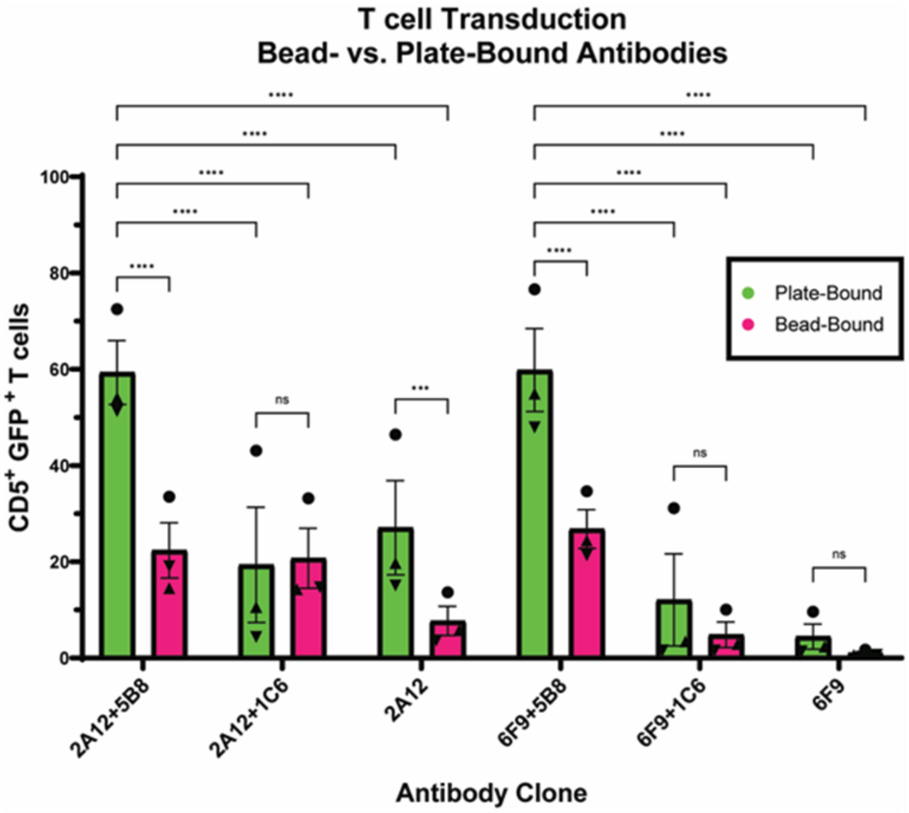

S9 Fig. Plate-bound αCD3 with 5B8 co-stimulation results in better transduction efficiency than bead-bound antibody. After three days of antibody-induced stimulation (6F9=CA17.6F9; 2A12=CA17.2A12), PBMCs from canine donors (n=3) were transduced with GFP gamma retrovirus. Frequency of GFP expressing $CD5^{+}$ T cells was evaluated three days after transduction. This figure contains the same data from Fig. 6 with focused statistical analysis on the effectiveness of antibody clones bound to plates or to beads. Horizontal lines indicate mean values, and error bars represent standard error of the mean. Multiple comparison statistical analysis was perfomed by Two-way ANOVA with Tukey's multiple means comparison; *** p<0.001, **** p<0.0001, ns not significant

7) Fig. S13. In the isotype group, the percentage of CD25+Foxp3+ cells appears unusually high at 17.76%. The authors should clarify whether this reflects high background staining or a gating issue. Additionally, in the 2A12+5B8 group, the percentage of CD25+Foxp3+ cells is reported as 9.76%; however, the total number of events in this plot appears markedly lower than in other groups.

We agree that the Treg population was high in the unstimulated isotype control group was high. However it was not anomalous (as is apparent in Figure 9). And as shown below in the minus CD25 and minus Foxp3 stain control figures, this is not attributable to gating issues or background staining.
As shown in Figure 7, the isotype control group in general fails to have substantial expansion in IL-2 enriched media that we attributed in the paper to the low frequency of CD25 expression in that group.
It would then follow that T cell subsets that constitutively express CD25, namely regulatory T cells, would survive better and proportionally increase over 14 days - supported by Moone et al. (Moon B-I, Kim TH, Seoh J-Y. Functional modulation of regulatory T cells by IL-2. PLoS One. 2015;10: e0141864.)

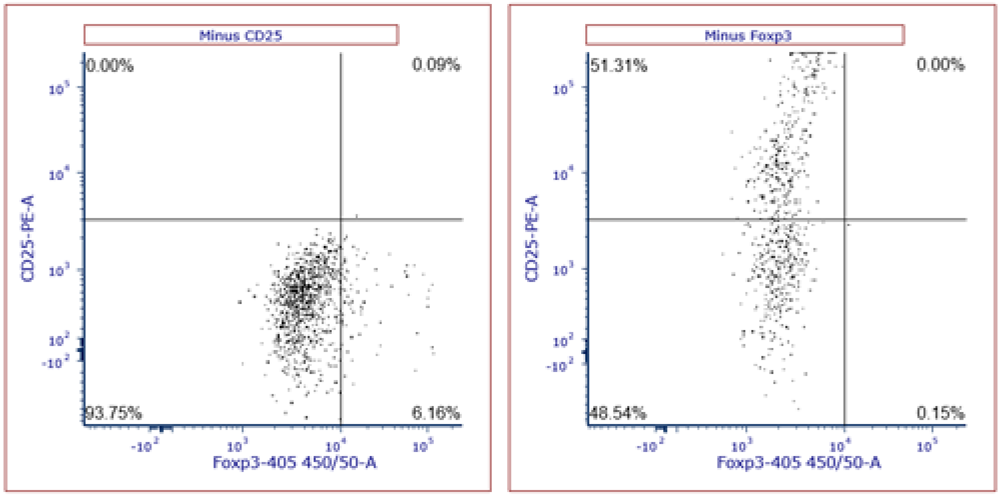

We added the following to the manuscript for elaboration:

> "Naive T cell populations have very low baseline CD25 expression. The constitutive CD25 expression in the Treg subpopulation was the most likely contributor to disproportionately higher expansion over time in IL-2 enriched media (Moon, Kim, and Seoh 2015), though the relative expansion compared to stimulated groups was quite modest."

Regarding the lower number of events with 2A12+5B8, this particular donor with this stimulation strategy had a lower number of CD4+ T cells in this sample than the others in the figure, which is the reason for the difference. While the event number is fewer, we felt there are enough events to see a clear delineation in proportionality. However, as the reviewer is requesting a figure with a higher number of events, we have replaced the data set with another as shown:

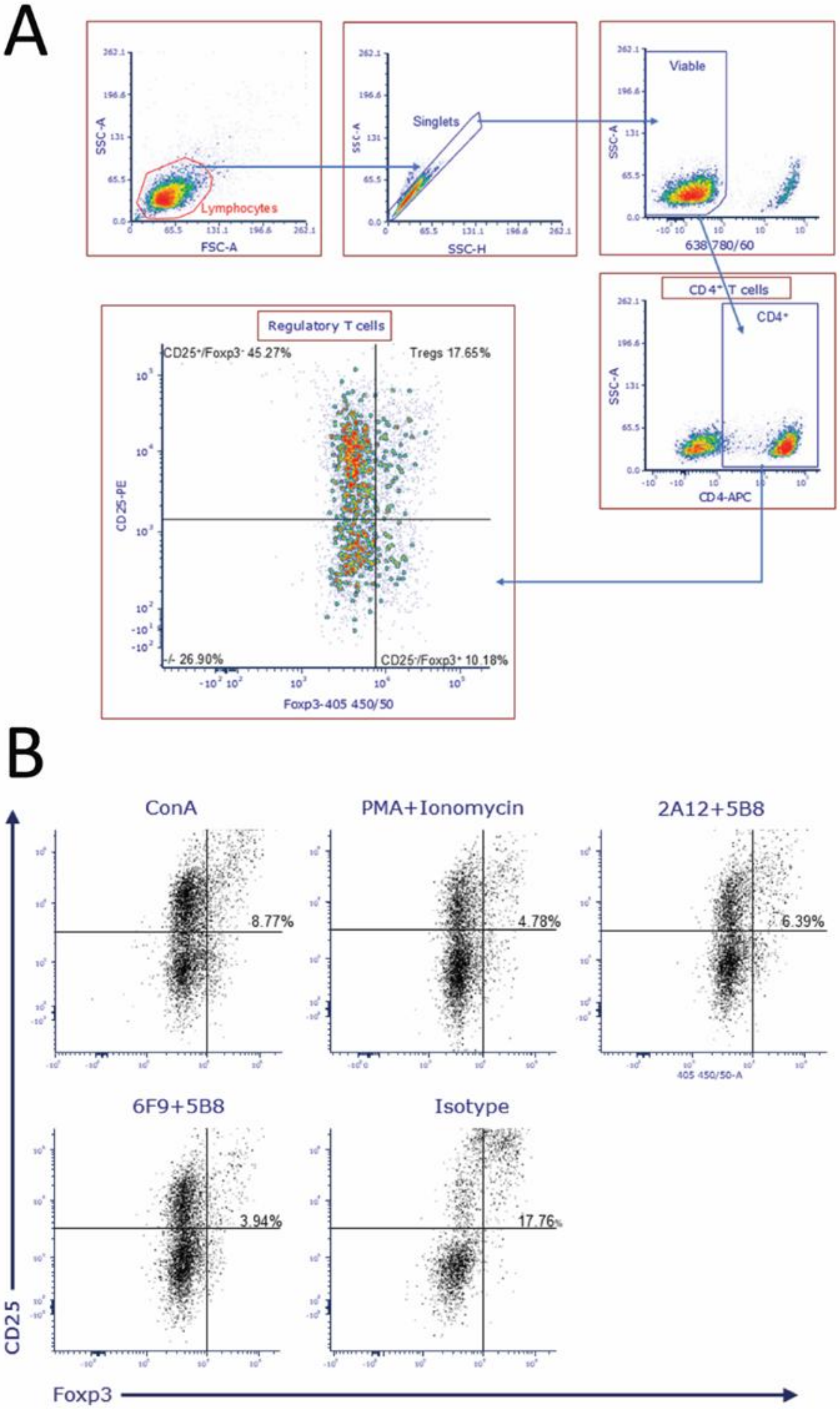
A
SSC-A
FSC-A
Lymphocytes
SSC-H
Singlets
Viable
638 780/60
CD4⁺ T cells
CD4⁺
CD4-APC
Regulatory T cells
CD25⁺/Foxp3⁻ 45.27%
Tregs 17.65%
-/- 26.90%
CD25⁻/Foxp3⁺ 10.18%
CD25-PE
Foxp3-405 450/50
B
ConA
8.77%
PMA+Ionomycin
4.78%
2A12+5B8
6.39%
405 450/50-A
6F9+5B8
3.94%
Isotype
17.76%
CD25
Foxp3

Reviewer #4: Yevgeny et al. conducted a valuable investigation into optimizing canine T cell activation, expansion, and transduction. While the study provides a solid foundational framework, additional rigorous and translational studies are necessary to substantiate the proposed mechanisms and enhance the clinical relevance of the findings. Incorporating the following elements would not only reinforce the central hypothesis but also significantly elevate the scientific impact and translational potential of the work.

Major Comments:
1. To deepen mechanistic insight and enhance translational relevance, further analysis of intracellular signaling pathways activated by different stimulation strategies—such as plate-bound 6F9+5B8 antibodies, PMA/ionomycin, or ConA—is highly recommended. This can be achieved through phospho-flow cytometry or Western blotting, focusing on key signaling molecules such as pZAP70, pAKT, and pSTAT5. Complementary transcriptomic profiling using bulk or single-cell RNA sequencing would help define T cell activation states, exhaustion signatures, and transduction susceptibility. Additionally, cytokine profiling via ELISA or Luminex could further elucidate functional polarization and cytokine secretion patterns.

We appreciate the reviewer's suggestions regarding mechanistic analysis and would like to ask for clarification regarding the feasibility and scientific rationale of the desired studies. We would appreciate the review's guidance on several issues.
Our search into commercially available antibodies reveals significant limitations for the proposed phospho-flow cytometry and Western blotting studies in canine samples:

- **pZAP70**: There is only one validated canine-reactive antibody available (Invitrogen MA5-15095), but no corresponding total ZAP70 antibody with confirmed canine reactivity. We are not sure that Western Blotting or "phospho flow cytometry" are feasible without both.
- **pAKT**: We could not find any antibodies validated for canine work. A few antibodies had predicted cross-reactivity based on sequence homology, but we worry about the time and expense it would take to validate multiple antibodies in canine cells.
- **pSTAT5**: No antibodies with confirmed canine validation found

In short, we worry these extensive studies are not feasible by the deadline requested by the editor (one month) and would greatly appreciate guidance from the reviewer on feasible ways to approach this important problem.
While single-cell RNA sequencing is possible in canine samples, we are concerned that implementing this approach would fundamentally transform our study from a targeted optimization investigation into a comprehensive mechanistic discovery project requiring specialized bioinformatics expertise. Our main concern is that our lab is simply not set up for such an undertaking and exploring this will substantially increase expenditures (~$50,000+ additional costs) and extend our timeline. Though we hope to expand our capabilities in the future, we ask for the reviewer's understanding of our current limitations and suggest that these important questions be addressed by others better equipped to do so in a timely manner.

2. To improve post-stimulation proliferation and gene transfer efficiency, a systematic evaluation of supportive cytokines—including IL-2, IL-7, IL-15, and IL-21—should be conducted to optimize T cell expansion while preserving desirable phenotypes. Further process optimizations such as spinoculation, retronectin-coated plates, serum-free culture conditions, and modified temperature regimens (e.g., transduction at 32–34 °C followed by expansion at 38.8 °C) may also enhance viral transduction and cell viability.

We appreciate this suggestion regarding additional cytokine optimization. Our study used IL-2 at concentrations established in prior canine T cell literature (200 U/mL during stimulation, 500 U/mL during expansion), achieving clinically relevant transduction rates (>70% with PMA/I, >50% with optimal antibody combinations). We agree that desirable phenotypes are very important for T cell persistence, especially as we approach clinical trials. We now highlight these important points in our DISCUSSION ,acknowledging that further optimization with IL-7 and IL-15 represents important future work to enhance memory T cell phenotypes. We also highlight the excellent work by Cieri et al. and preliminary canine studies by Panjwani et al. who have already explored this question. In addition, we highlight IL-21 with IL-2 and IL-15 have been evaluated historically, by Sakai et al. and we have added this to our DISCUSSION as follows:

> "Cieri [92] demonstrated that the presence of IL-7 and IL-15 in culture promotes survival and expansion of human T cells while promoting a less differentiated TSCM memory phenotype. This has also been demonstrated in canine T cells to some degree by Panjwani [5], who demonstrated cross reactivity of human recombinant IL-7 and IL-15 in producing less differentiated T cells…Sakai explored canine T cell expansion with various combinations of IL-2, IL-15, and IL-21. Though the addition of these cytokines drove more reliable expansion in PHA stimulated T cells with less variability, they failed to produce a substantial proportion of TSCM cells and resulted in predominantly TEFF cell subset differentiation [12]. Further experiments are necessary to fully understand how IL-2, IL-7, IL-15, and IL-21 influence the repertoire of canine T cell subsets."

We intend to dive more deeply into cytokine influence on T cell differentiation in future experiments. However, as others have already published good data on this question, we believe that limiting the study scope to optimizing stimulation strategies in the context of activation, expansion, and transduction is appropriate. We feel that our current results meet clinical benchmarks for CAR-T manufacturing and we believe cytokine optimization studies would be more appropriately addressed in dedicated follow-up investigations.

3. Long-term functional validation is critical. Incorporating cytotoxicity assays—such as chromium release or LDH assays—using relevant canine tumor cell lines would directly assess the anti-tumor efficacy of the genetically modified T cells. Concurrently, longitudinal flow cytometric analysis of memory markers (CD62L, CCR7) and exhaustion markers (PD-1, LAG-3, TIM-3) would provide insights into the persistence, functional status, and therapeutic durability of the expanded T cell populations.

We appreciate the reviewer's comments about the need for functional validation. We respectfully note that our study used reporter virus to optimize T cell stimulation and transduction conditions, but did not generate functional CAR-T cells with tumor specificity. GFP-transduced cells lack antigen recognition capability, making cytotoxicity assays scientifically premature. As we acknowledge in our discussion, '**transduction experiments utilized GFP gamma retrovirus and evaluated GFP expression as a marker for successful transduction,**' with the caveat that functional CAR expression may differ.

With respect to the suggested longitudinal flow cytometric analyses, they are scientifically valuable, but we feel that they will need to be done with CAR T cells and in the context of CAR efficacy rather than manufacturing optimization.

We believe our work provides the essential foundation for future functional CAR-T studies by establishing optimal stimulation protocols that achieve clinically relevant transduction rates (>70% with PMA/I, >50% with antibody combinations)."

We are excited to share - confidentially - that we are well on our way to optimizing viral vector design for a B7H3-targeting canine CAR T cell, which included “caninized” co-activation domains. Although the work is not quite ready for publication, we hope to submit it soon.

The reviewer’s comment is especially important for future work. We direct the reviewer to this portion of our DISCUSSION section where we will specifically address the functionality, durability, and persistence of these CAR T cells in our next publication:

> “In future work we plan to use plate-bound 6F9+5B8 and PMA/I-induced transduction methods to produce tumor antigen-specific CAR T cells. In a cytotoxicity assay, we will assess how these different transduction strategies impact CAR T cell functionality and persistence by evaluating target cell killing, cytokine production, T cell exhaustion, and memory differentiation.”

4. Investigation into canine-specific viral restriction factors such as SAMHD1 and APOBEC3, and the use of small molecule inhibitors or siRNA-mediated knockdown strategies, may further improve retroviral gene transfer efficiency. Moreover, the application of epigenetic modulators, including HDAC or DNA methyltransferase inhibitors, could enhance transgene expression and stability. Together, these mechanistic and translational strategies would not only strengthen the therapeutic foundation of canine T cell immunotherapy but also offer valuable insights for the development of comparable approaches in human cancer immunotherapy.

Thank you for your thoughtful comment regarding viral restriction factors. We appreciate you highlighting the importance of SAMHD1 and APOBEC3, which are indeed well-established and critical viral restriction factors in human biology. SAMHD1 functions as a dNTP triphosphohydrolase that restricts retroviral replication in non-cycling cells, while APOBEC3 family members are cytidine deaminases that restrict various DNA and RNA viruses through mutagenic and non-mutagenic mechanisms.

We would like to respectfully get further clarity for the reviewer as our understanding of canine biology seems to differ from the reviewer’s and would like to better understand what the reviewer refers to as "canine-specific viral restriction factors." An extensive literature search revealed limited evidence for the functional role of these proteins in canine biology. While dogs possess three APOBEC3 genes compared to seven in humans (Caval et al. https://doi.org/10.1093/molbev/mst195), and evolutionary homology suggests canine SAMHD1 orthologs exist (Briggs et al. https://doi.org/10.1371/journal.pone.0017107), we found no studies demonstrating endogenous expression or functional activity of these restriction factors in canine T cells. The only relevant studies we identified (Zhangming et al. https://doi.org/10.1080/15384101.2022.2085356) involved ectopic expression of human or murine SAMHD1 in Madin-Darby canine kidney (MDCK) cells, which does not establish the natural role of these factors in canine immune cells.

Could you please clarify what specific evidence supports the significance of these restriction factors in canine T cell biology, particularly in the context of retroviral transduction? If such evidence exists, we would be grateful for the references.

Additionally, our study achieved clinically relevant transduction rates (>70% with PMA/I stimulation, >50% with optimal antibody combinations) using single-round transduction, which compares favorably to previous reports and meets the threshold for therapeutic applications. Given these already high transduction efficiencies, we worry that further optimization through restriction factor manipulation may not be necessary for the clinical objectives of canine CAR-T cell therapy.

We are currently engaged in a detailed and robust process to identify conditions and factors involved in viral transduction with a CAR-encoding virus. We are hopeful that as we progress in this investigation we can meet the reviewer's request, but hope that this kind of extensive and time-consuming study can be left for our next publication. We value the reviewer's thoughts on the matter.
